# Unveiling the ZNF384-INTS13-hnRNPC axis as a therapeutic vulnerability in cervical cancer

**DOI:** 10.1038/s41419-025-08374-6

**Published:** 2025-12-22

**Authors:** Juan Wang, Shuang Liu, Ping Li, Yasser Perera Negrin, Fang-Rong Shen, Rong Ma

**Affiliations:** 1 Department of Obstetrics and Gynecology, the First Affiliated Hospital of Soochow University, Suzhou, China; 2 Department of Gynecology, Harbin Medical University Cancer Hospital, Harbin, China; 3 Department of Radiotherapy and Oncology, Affiliated Kunshan Hospital of Jiangsu University, Kunshan, China; 4 Research Department, China-Cuba Biotechnology Joint Innovation Center, Yongzhou, China; 5 Department of Pharmaceuticals, Center for Genetic Engineering and Biotechnology, Havana, Cuba

**Keywords:** Cervical cancer, Oncogenes

## Abstract

Cervical cancer remains a major global health burden, necessitating the identification of novel therapeutic targets to overcome the limitations of current treatments. Here, we comprehensively investigated the role of integrator complex subunit 13 (INTS13) in cervical cancer progression. Our analysis of publicly available The Cancer Genome Atlas (TCGA) datasets revealed that *INTS13* is significantly overexpressed in cervical cancer tissues across various histological subtypes, correlating with advanced tumor T-stage and predicting poorer overall survival. Single-cell RNA sequencing further localized INTS13 expression predominantly to malignant epithelial cells within the tumor microenvironment, where its expression correlated with genes involved in critical cellular processes. Furthermore, elevated expression has been observed in cervical cancer tissues from surgically-treated patients and in various primary human cervical cancer cells. In vitro functional studies demonstrated that genetic silencing or CRISPR/Cas9-mediated knockout of INTS13 significantly inhibited the proliferation, migration, and invasion of primary cervical cancer cells, while selectively inducing apoptosis. Conversely, ectopic INTS13 overexpression markedly enhanced these malignant phenotypes. Mechanistically, we identified heterogeneous nuclear ribonucleoprotein C (hnRNPC) as a critical downstream effector, with INTS13 regulating hnRNPC expression, and the restoration of hnRNPC effectively reversing the anti-cervical cancer effects observed upon INTS13 silencing. Furthermore, the transcription factor ZNF384 (zinc finger protein 384) was identified as an upstream regulator that directly binds to and positively governs INTS13 expression. Finally, in vivo animal models confirmed that targeted silencing of INTS13 significantly impeded cervical cancer xenograft growth in nude mice, reduced cellular proliferation, and augmented apoptosis, consistently accompanied by a reduction in hnRNPC expression. These findings collectively establish INTS13 as a crucial precancerous gene in cervical cancer, promoting malignant phenotypes primarily through the ZNF384-INTS13-hnRNPC signaling axis.

## Introduction

Human cervical cancer continues to represent a formidable global health challenge [1], standing as the fourth most frequently diagnosed cancer and the fourth leading cause of cancer-related death among women worldwide [2, 3], with an estimated over 600,000 new cases and 340,000 deaths annually [2, 3]. Cervical carcinoma exhibits diverse histological subtypes, primarily categorized into squamous cell carcinoma (SCC) and adenocarcinoma (AC), with distinct epidemiological profiles and clinical implications. SCC constitutes the vast majority, typically accounting for 80–90% of cases and originating from the ectocervix, often within the transformation zone [4, 5]. AC, representing 10–20% of cases, arises from the glandular epithelium of the endocervix [1, 6]. Despite substantial advancements in preventative strategies, including widespread screening programs and the application of human papillomavirus (HPV) vaccination [7], its prevalence, particularly within low- and middle-income countries, persists as a critical public health issue [1, 6].

HPV is unequivocally established as the primary causative agent of virtually all cervical cancer cases [1, 8]. Specifically, persistent infection with high-risk HPV types, such as HPV16 and HPV18, is the critical event that initiates the process of cervical carcinogenesis [1, 8]. These viruses encode oncogenes, including E6 and E7, which interfere with key tumor suppressor proteins in host cells. For example, the HPV E6 oncoprotein promotes the degradation of p53, a protein vital for DNA repair and apoptosis, thereby allowing damaged cells to survive and proliferate [9, 10]. Concurrently, the E7 oncoprotein binds to and inactivates the retinoblastoma protein (pRb), leading to uncontrolled cell division and growth [11, 12]. This combined disruption of cellular regulatory pathways creates a permissive environment for the accumulation of genetic mutations, ultimately leading to the malignant transformation of cervical epithelial cells [11]. The prognosis for patients diagnosed with advanced or recurrent cervical cancer remains suboptimal, as current standard-of-care treatment modalities, primarily encompassing radical surgery, radiotherapy, and systemic chemotherapy, often yield limited long-term efficacy [13, 14]. A fundamental limitation of these conventional therapeutic approaches lies in their inherent lack of specificity, frequently resulting in considerable systemic toxicity, such as myelosuppression, nephrotoxicity, and gastrointestinal disturbances, alongside the pervasive challenge of acquired treatment resistance [1, 15, 16].

The therapeutic landscape for cervical cancer has progressively evolved with the emergence of targeted molecular therapies [1, 17–19]. These innovative approaches are designed to precisely interfere with specific molecular pathways or proteins that are indispensable for the proliferation, survival, and metastatic potential of cancer cells [17–19]. Such therapies offer the compelling promise of enhanced therapeutic efficacy coupled with a more favorable side-effect profile when compared to traditional cytotoxic chemotherapy [17–19]. However, despite their undeniable benefits, current targeted agents for cervical cancer, such as the anti-angiogenic agent bevacizumab or immune checkpoint inhibitors like pembrolizumab, are often constrained by limitations [17–19]. These include the development of both primary and acquired resistance mechanisms, the inherent heterogeneity in patient response, and the ongoing challenge of identifying robust predictive biomarkers for optimal patient stratification [1, 17–19]. Consequently, the urgent imperative to discover and validate novel molecular targets for more effective therapeutic intervention in cervical cancer remains a paramount research priority [1, 17–19].

The Integrator complex is a multi-subunit ribonucleoprotein assembly fundamentally involved in the 3’-end processing of small nuclear RNAs (snRNAs) [20–22]. This critical function is essential for the biogenesis and stability of snRNAs, which in turn play a vital role in pre-mRNA splicing [20–22]. Beyond its well-established role in RNA processing, accumulating evidence indicates that various components of the Integrator complex participate in a broader spectrum of cellular processes, including the sophisticated regulation of gene expression, responses to DNA damage, control of cell cycle progression, and mechanisms of mRNA degradation [23, 24]. Here, a comprehensive differential expression analysis of all known Integrator complex subunits was performed using data from The Cancer Genome Atlas (TCGA) and public single-cell RNA (scRNA) sequencing datasets to identify those significantly upregulated in cervical cancer tissues/cells. Next, subunits exhibiting a strong correlation with poor patient survival outcomes were prioritized. Finally, a thorough literature review was conducted to exclude any subunits with already well-established roles in malignancies. This rigorous approach ultimately led to the selection of INTS13 as a novel and clinically relevant target for detailed study in cervical cancer.

The precise functional role and detailed mechanism of action of INTS13, particularly its potential direct association with the pathogenesis of human diseases, including various malignancies, have yet to be comprehensively elucidated. In this study, our primary goal was to comprehensively investigate the functional role and underlying mechanisms of INTS13 in cervical cancer. We pursued this by first analyzing its expression and clinical relevance using publicly available datasets and patients’ tissues. We then performed a detailed functional characterization of INTS13, exploring its impact on malignant phenotypes both in vitro and in vivo. Finally, we elucidated the key molecular signaling axis that governs INTS13’s pro-cancerous effects, identifying both an upstream transcription factor (ZNF384) and a critical downstream effector (hnRNPC).

## Materials and methods

### Reagents and antibodies

Reagents and antibodies were obtained from: Sigma-Aldrich (Puromycin, cell culture media, FBS, Matrigel matrix, polybrene, CCK-8 and caspase inhibitors), Thermo-Fisher Scientific (TUNEL, EdU, DAPI and JC-1 dyes), Cell Signaling Technology and Thermo Fisher Scientific (primary antibodies). Nucleotide sequences and PCR primers were synthesized and verified by Genechem.

### Cells

As reported previously [25], primary human cervical cancer cells (pCCa-1, -2, -3, from three informed SCC patients) and cervical epithelial cells (priCEpi-1, -2, from two patients) were isolated from fragmented cervical cancer and adjacent epithelial tissues via collagenase type I and dispase II digestion (Sigma), followed by standard processing and cultivation in complete medium [26]. HeLa cervical cancer cell line was obtained from the Cell Bank of the Institute of Biological Science at CAS (Shanghai, China). All human cell procedures adhered to the Helsinki Declaration and were approved by the Ethics Committee of The Affiliated Kunshan Hospital of Jiangsu University (JDKY-2022-135#).

### Human tissues

Cervical cancer and corresponding paracancerous epithelial tissues (*n* = 20) were collected from patients at the Affiliated Hospitals of Soochow University, with informed consent obtained. Patient details are previously reported [26]. All procedures adhered to the Helsinki Declaration and were approved by the Ethics Committee of The Affiliated Kunshan Hospital of Jiangsu University (JDKY-2022-135#).

### Bioinformatic analysis of the TCGA dataset

We obtained RNA-sequencing (RNA-seq) and clinical data from the TCGA-CESC project. The RNA-seq count matrix was normalized to Transcripts Per Million values. The study cohort included 35 adjacent cervical tissues and 309 tumor tissues, which were histologically classified as (SCC, *n* = 255), (AC, *n* = 47), and adenosquamous carcinoma (ASC, *n* = 7). Clinical data, including TNM stage, BMI (Body Mass Index), Overall Survival (OS), and Disease-Specific Survival, were collected for each patient. For survival analysis, patients were dichotomized into *INTS13*-high and *INTS13*-low expression groups based on an optimal cut-off determined by the surv_cutpoint function in the “survminer” R package.

### Single-cell RNA sequencing analysis

Single-cell RNA sequencing (scRNA-seq) data from cervical cancer samples (ArrayExpress, accession number E-MTAB-11948) were processed using the Seurat analysis pipeline. This dataset, published by Li et al., [27] includes samples from three cervical SCC patients (tumor and adjacent tissues). The total number of profiled cells was 57,669. After a rigorous quality control process, 6569 low-quality cells were excluded. The number of cells from each patient included in the final analysis was 20,655, 17,810, and 12,635, respectively [27]. Following quality control, the data were normalized to correct for sequencing depth differences. The top 2000 most variable genes were identified and scaled. Principal Component Analysis was then performed for dimensionality reduction, using significant components for subsequent steps. For cell type identification, graph-based clustering was performed using the FindNeighbors and FindClusters functions with an optimized resolution. The resulting clusters were visualized with uniform manifold approximation and projection (UMAP) and manually annotated using established marker genes. The epithelial cell subpopulation was extracted, and cells expressing the *INTS13* gene were identified. A gene co-expression analysis was performed within this group. The top 100 genes most positively correlated with *INTS13* were selected. Finally, functional and pathway enrichment analysis of these genes was conducted using the Enrichr tool.

### Immunohistochemistry (IHC)

As reported previously [25], IHC was performed on 4 μm paraffin-embedded tissue sections. After de-paraffinization and rehydration, antigen retrieval was utilized with citrate buffer. Endogenous peroxidase was inhibited with hydrogen peroxide. Primary antibody incubation was 12 h, followed by 1.5 h incubation with secondary antibody and streptavidin-horseradish peroxidase conjugate. Target antigen expression was visualized using diaminobenzidine.

### Western blotting

As reported previously [25], 30–40 µg protein extracts from cellular and tissue lysates were resolved on 8–12% SDS-PAGE gels, transferred to PVDF membranes, blocked, and incubated with primary antibodies overnight at 4 °C, then secondary antibodies for 45 min at room temperature. Protein bands were visualized by enhanced chemiluminescence and quantified with ImageJ. All uncropped blotting images are listed in Fig. S1.

### qPCR

As reported previously [25], RNA was isolated from cells and tissues (TRIzol, Biyuntian), reverse transcribed into cDNA (Takara PCR amplification kit), and quantified by qPCR (SYBR Green PCR Master Mixes) on an ABI-7900 system. *GAPDH* served as the reference gene; relative gene expression was determined by the 2^–ΔΔCt^ method. The verified primers were from GenePharm.

### Gene silencing

INTS13 gene silencing utilized three distant shRNAs (shINTS13-Sq1, shINTS13-Sq2, and shINTS13-Sq3, each targeting nonoverlapping sequences against human *INTS13*) sub-cloned into the GV369 lentiviral vector (Genechem, as reported previously [25]). Lentiviral particles, generated by transfecting constructs with packaging plasmids into HEK-293 cells, were transduced into cervical cancer or epithelial cells. Stable INTS13 knockdown cells were established via puromycin selection and validated by qPCR and Western blotting. Silencing ZNF384 (zinc finger protein 384) was through the same procedure and targeting specific sequences of ZNF384 based on Dr. Cao [28]: shZNF384-Sq1; shZNF384-Sq2.

### *INTS13* gene knockout

*INTS13* gene knockout in primary cervical cancer cells involved culturing cells to 60% confluence in complete medium with polybrene, then transducing with a Cas9 nuclease-expressing lentiviral vector (reported previously [25]) to generate stable cells. These cells were further transduced with two CRISPR/Cas9 constructs containing unique and verified sgRNA sequences (koINTS13-sg1 and koINTS13-sg2) for INTS13 knockout. Following puromycin selection, single-cell clones were isolated. Successful INTS13 knockout was validated by DNA sequencing and Western blotting. Control cells (“koC”) were generated by transducing with the Cas9 lentiviral vector and a non-targeting scrambled control sgRNA (reported previously [25]).

### Gene overexpression

INTS13 overexpression used the hINTS13-expressing lentiviral GV369 construct (Genechem). Lentiviral particles, produced by transfecting this construct with packaging plasmids into HEK-293 cells, were employed to infect cultured cervical cancer cells. Cells were then transferred to puromycin-containing medium after 48 h. Stable INTS13-overexpressing cells were established after five to six passages, with overexpression validated by qPCR and Western blotting. Overexpression of hnRNPC was via the same procedure. Overexpression of ZNF384 was using the lentiviral construct provided by Dr. Yin [29]. After puromycin treatment and selection for five to six passages, ZNF384 overexpression was confirmed using qPCR and Western blotting.

### CCK-8 assay

Cell viability was assessed using the CCK-8 method. As reported previously [29], 3000 cells/well were seeded in 96-well plates for 96 h. Subsequently, 15 μL of CCK-8 solution was added per well and incubated for 95 min. Absorbance at 435 nm was measured with a microplate reader to determine viability.

### Colony formation assay

As reported previously [29], 12,000 genetically modified cervical cancer cells were seeded per 10 cm dish. After 10 days, colonies were stained and manually counted.

### Nuclear EdU/TUNEL staining

As reported previously [29], the genetically modified cervical cancer cells or epithelial cells, cultured in 12-well plates, were fixed with paraformaldehyde and permeabilized with Triton X-100. Cells were stained with TUNEL/EdU and DAPI dyes, then imaged using a Zeiss fluorescence microscope. The ratio of EdU/TUNEL-positive cells to DAPI-stained nuclei was quantified.

### Transwell assay

The detailed procedures were reported previously [29]. In brief, Transwell assays utilized 10,000 cervical cancer cells/condition, seeded on the upper insert of the Transwell chambers for 24 h. Migrated cells on the lower surface were fixed and crystal violet-stained. For invasion assays, inserts were always Matrigel-coated.

### JC-1 staining

Mitochondrial membrane potential (MMP) was assessed using an Invitrogen JC-1 assay kit per manufacturer guidelines. In brief, genetically modified cervical cancer cells were incubated with 3.0 μg/mL JC-1 dye for 20 min, followed by warm PBS washes. Reduced MMP was indicated by green fluorescence (monomers), measured at 475 nm excitation via fluorescence microscopy.

### Cytosolic cytochrome c detection

Cytosolic Cytochrome c levels were quantified by ELISA (Thermo-Fisher Invitrogen, Suzhou, China). The assay employed Cytochrome c-specific capture antibodies immobilized on a microplate. Cytosolic protein lysates (1.0 μg/μL, 25 μg/sample) were added, allowing binding. An enzyme-linked detection antibody formed a complex, followed by substrate addition for a colorimetric reaction, detected at 450 nm. A standard curve was determined for cytosol Cytochrome c levels.

### Caspase activity assessment

The detailed procedures were reported previously [29]. In brief, Caspase-3 and Caspase-9 activities were assessed using colorimetric assay kits (Thermo-Fisher Invitrogen, Suzhou, China) per manufacturer's protocols. Cell lysates (1.0 μg/μL, 25 μg/sample) were incubated with specific caspase substrates. Enzymatic cleavage yielded a colorimetric product, spectrophotometrically measured at 415 nm. A standard curve determines caspase activity.

### Chromatin immunoprecipitation (ChIP)

ChIP assay followed the protocol outlined in an early publication [29]. Initially, fresh tissue or total cellular material was lysed and homogenized. These lysates were then diluted in ChIP dilution buffer, a reagent kindly provided by Dr. Yin [29]. Subsequently, the samples underwent immunoprecipitation using an anti-ZNF384 antibody (also provided by Dr. Yin [29]). The DNA fragments bound to ZNF384 were then isolated by elution from protein A/G agarose (Santa Cruz Biotech) containing NaCl. Finally, the enrichment of the predicted *INTS13* promoter sequence, identified through the JASPAR database with the highest score, was subsequently quantified by qPCR.

### Xenograft assays

The detailed procedures were reported previously [30]. Female BALB/c nude mice (17.9–18.3 g), acquired from Shanghai SLAC Laboratory Animal Co. (Shanghai, China), served as xenograft models [25]. Each mouse received a subcutaneous injection of seven million genetically modified primary cervical cancer cells into the flank. Tumor volumes, body weights, and daily growth rates (mm^3^/day) were monitored starting 21 days post-inoculation, adhering to established guidelines [25]. Apoptosis within tumor sections was assessed via TUNEL fluorescence staining (Biyuntian kit, Wuxi, China), followed by DAPI counterstaining for nuclear visualization. All animal procedures were approved by the Institutional Animal Care and Use Committee (IACUC) and the Ethics Review Board of The Affiliated Kunshan Hospital of Jiangsu University (JDKY-2022-135#).

### Statistical analysis

In vitro experiments were performed with five biological replicates (*n* = 5). Normally distributed data were always presented as mean ± standard deviation. Unpaired Student’s *t* tests compared two experimental groups. For three or more groups, one-way ANOVA was used, with post-hoc Scheffé's and Tukey’s multiple comparison tests. Statistical significance was set at *P* < 0.05.

### Ethics approval and consent to participate

All methods adhered to relevant guidelines and regulations. Participants provided written informed consent for the use of their human tissues and primary human cells. The management of these human tissue samples and cells followed protocols by the Ethics Committee of the Affiliated Kunshan Hospital of Jiangsu University (JDKY-2022-135#) and was conducted in strict adherence to the ethical guidelines outlined in the Declaration of Helsinki. All animal procedures were approved by the IACUC and the Ethics Review Board of The Affiliated Kunshan Hospital of Jiangsu University (JDKY-2022-135#).

## Results

### Elevated *INTS13* expression predicts poor prognosis in cervical cancer and correlates with key clinicopathological features

**Fig. 1:**
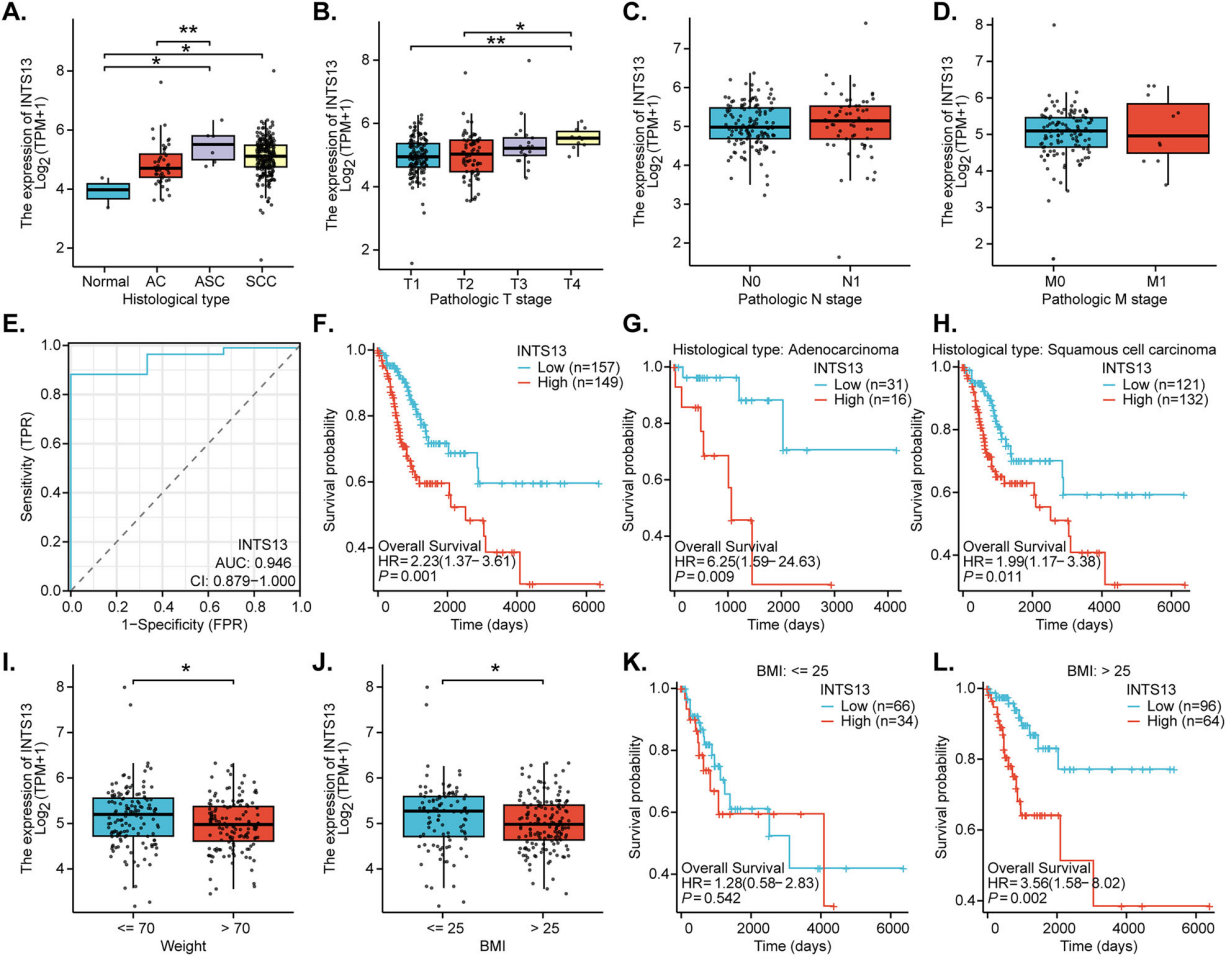
Elevated *INTS13* expression predicts poor prognosis in cervical cancer and correlates with key clinicopathological features. Box plots illustrating the differential expression of *INTS13* in cervical tissues versus various histological subtypes of cervical cancer (adenocarcinoma [AC], adenosquamous carcinoma [ASC], and squamous cell carcinoma [SCC]) based on TCGA data (**A**). Box plots showing the expression levels of *INTS13* across different T-stages, N-stages, and M-stages within the TCGA cervical cancer cohort (**B**–**D**). A receiver operating characteristic (ROC) curve demonstrating the diagnostic performance of *INTS13* for cervical cancer in the TCGA dataset (**E**). Kaplan-Meier overall survival curves comparing patients with high versus low *INTS13* expression across the entire TCGA cervical cancer cohort (**F**), or within the adenocarcinoma (AC) (**G**) and squamous cell carcinoma (SCC) (**H**) histological subtypes from the TCGA dataset. Box plots illustrating the relationship between *INTS13* expression and patient body weight categories from TCGA data (**I**, **J**). Kaplan-Meier overall survival curves comparing patients with high versus low *INTS13* expression stratified by body weight subgroups from the TCGA dataset (**K**, **L**). “TPM” stands for transcripts per million. “AUC” stands for area under the curve. “CI” stands for confidence interval. “HR” stands for hazard rate. “TPR*”* stands for true positive rate. “FPR*”* stands for false positive rate. “BMI” stands for body mass index. Data are presented as mean ± standard deviation (SD). * *P* < 0.05. ** *P* < 0. 01.

We first derived publicly available data from TCGA cohort. As depicted in Fig. 1A, the expression of *INTS13* was found to be significantly elevated within cervical cancer tissues when compared to adjacent cervical tissues. Further stratification by histological subtype revealed consistently high expression of *INTS13* across AC, SCC, and ASC, with SCC exhibiting the most pronounced upregulation (Fig. 1A). Investigation into the correlation between *INTS13* expression and clinicopathological staging, specifically the TNM classification (Fig. 1B–D), indicated a statistically significant progressive increase in *INTS13* expression with advancing T-stage (Fig. 1B). Conversely, no statistically significant differences in *INTS13* expression were observed in relation to N-stage (regional lymph node metastasis) (Fig. 1C) or M-stage (distant metastasis) (Fig. 1D). The diagnostic utility of *INTS13* for cervical cancer was rigorously assessed through receiver operating characteristic curve analysis (Fig. 1E), which yielded a high area under the curve value, indicative of its strong potential as a diagnostic biomarker for distinguishing cervical cancer from peritumoral tissue.

Subsequent OS analyses, based on TCGA cervical cancer data, revealed that the expression level of *INTS13* significantly impacted the prognosis of cervical cancer patients (Fig. 1F–H). Figure 1F illustrated the OS outcomes for the entire cohort of patients with cervical cancer, demonstrating that individuals with elevated *INTS13* expression exhibited significantly poorer OS relative to those with low expression. The optimal cut-off for dividing patients into “high” and “low” expression groups was determined using the surv_cutpoint function from the survminer R package. This function selects the best cut-off value that minimizes the *P*-value from a log-rank test, ensuring the most significant separation between the two groups. Subgroup analyses were undertaken for distinct histological subtypes: Fig. 1G depicts the OS curves for patients with AC, whereas Fig. 1H presents the corresponding data for those with SCC. In both subtypes, high *INTS13* expression was correlated with a diminished prognosis, consistent with the pattern observed across the overall cohort (Fig. 1F). Furthermore, a significant inverse association was observed between *INTS13* expression and patient body weight (Fig. 1I, J), where individuals with higher body weight tended to exhibit lower *INTS13* expression levels. To explore this relationship further, a subgroup analysis of OS data was performed based on body weight categories (Fig. 1K, L). This analysis revealed that the prognostic impact of differential *INTS13* expression (high versus low) on cervical cancer survival was particularly prominent and statistically significant within the higher body weight subgroup (BMI > 25), where high *INTS13* expression correlated with markedly reduced survival (Fig. 1L). Conversely, in patients with lower body weight (BMI ≤ 25), the difference in OS between high and low *INTS13* expression groups did not reach statistical significance (Fig. 1K). Therefore, the TCGA-based analysis shows that *INTS13* is overexpressed in cervical cancer, correlates with tumor T-stage, exhibits high diagnostic potential, and serves as an independent prognostic marker for poorer OS in cervical cancer patients, particularly those with higher body weight.

### Single-cell analysis of *INTS13* expression and functional associations in cervical squamous cell carcinoma

**Fig. 2:**
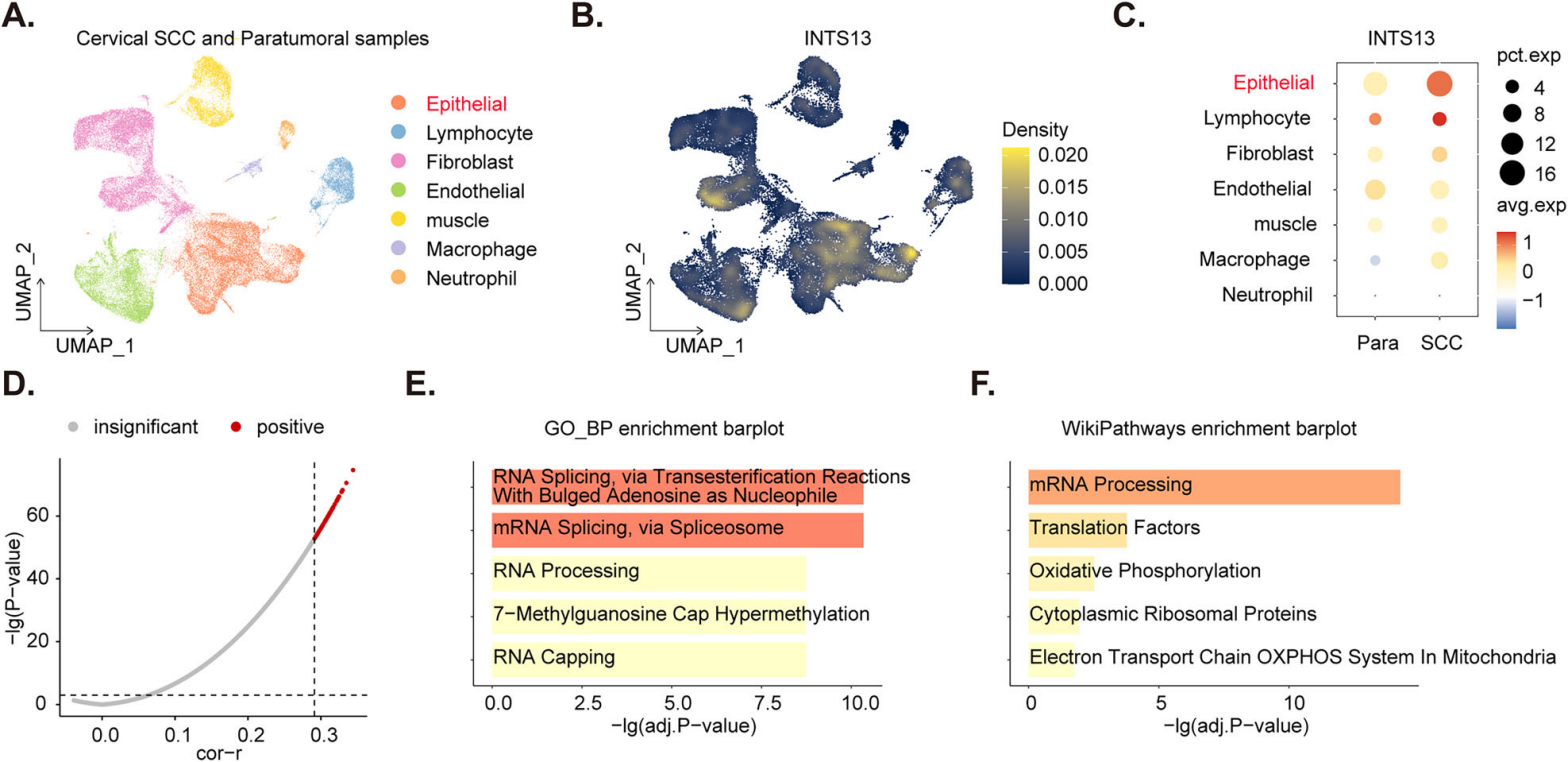
Single-cell analysis of *INTS13* expression and functional associations in cervical squamous cell carcinoma. The uniform manifold approximation and projection (UMAP) plot displaying the clustering and annotation of distinct cell populations (epithelial, lymphocyte, fibroblast, endothelial, muscle, macrophage, and neutrophil) identified from single-cell RNA sequencing data of cervical squamous cell carcinoma (SCC) and paracancerous samples (ArrayExpress accession E-MTAB-11948) (**A**). The UMAP plot illustrating the expression density of *INTS13* across the identified cell clusters (**B**). The Dot plot quantifying the average expression levels and percentage of cells expressing *INTS13* within specific cell populations in SCC samples compared to paracancerous tissues (**C**). The Scatter plot depicting the correlation coefficients (cor-r) and negative log10 *P*-values for genes correlated with *INTS13* expression in epithelial cells, highlighting the top 100 positively correlated genes selected for further analysis (**D**). A Gene Ontology Biological Process (GO_BP) enrichment bar plot displaying the most significantly enriched biological processes associated with the top 100 *INTS13*-correlated gene set (**E**). A WikiPathways enrichment bar plot showcasing the most significantly enriched pathways associated with the top 100 *INTS13*-correlated gene set (**F**).

To precisely delineate the cellular localization and functional associations of *INTS13* at a single-cell resolution, we conducted an in-depth analysis of scRNA sequencing data derived from cervical SCC samples, as SCC accounts for 80–90% of all cervical cancer cases [4, 20]. This dataset was retrieved from the ArrayExpress database, specifically under accession number E-MTAB-11948. As comprehensively illustrated by the UMAP plot in Fig. 2A, the diverse cellular populations comprising the cervical SCC tumor microenvironment were successfully and distinctly annotated. These populations included epithelial cells, lymphocytes, fibroblasts, endothelial cells, muscle cells, macrophages, and neutrophils (Fig. 2A). An examination of the expression density plot for *INTS13* (Fig. 2B) unequivocally demonstrated that *INTS13* exhibited a predominant expression profile within the epithelial cell cluster. Furthermore, a quantitative assessment presented as a dot plot (Fig. 2C) revealed a significant upregulation of *INTS13* expression within the epithelial cell populations in SCC samples when directly compared to their corresponding paracancerous tissue counterparts (Fig. 2C).

To systematically identify genes co-expressed with *INTS13* and subsequently predict their potential biological functions, a comprehensive correlation analysis was performed, focusing on *INTS13* expression specifically within the malignant epithelial cell population. From this analysis, the top 100 genes exhibiting the highest correlation with *INTS13* expression were carefully selected and designated as INTS13-associated genes (Fig. 2D). Subsequent functional and pathway enrichment analyses of these identified *INTS13*-associated genes (Fig. 2E, F) demonstrated significant enrichment in critical cellular processes. These included, but were not limited to, RNA splicing, RNA processing, oxidative phosphorylation (OXPHOS), and components of the mitochondrial electron transport chain (Fig. 2E, F). These findings suggest that *INTS13* may exert a pivotal regulatory influence over these fundamental cellular activities within the intricate pathological landscape of cervical SCC cancer cells.

### Elevated INTS13 expression in surgically-treated cervical cancer tissues and various cervical cancer cell types

**Fig. 3:**
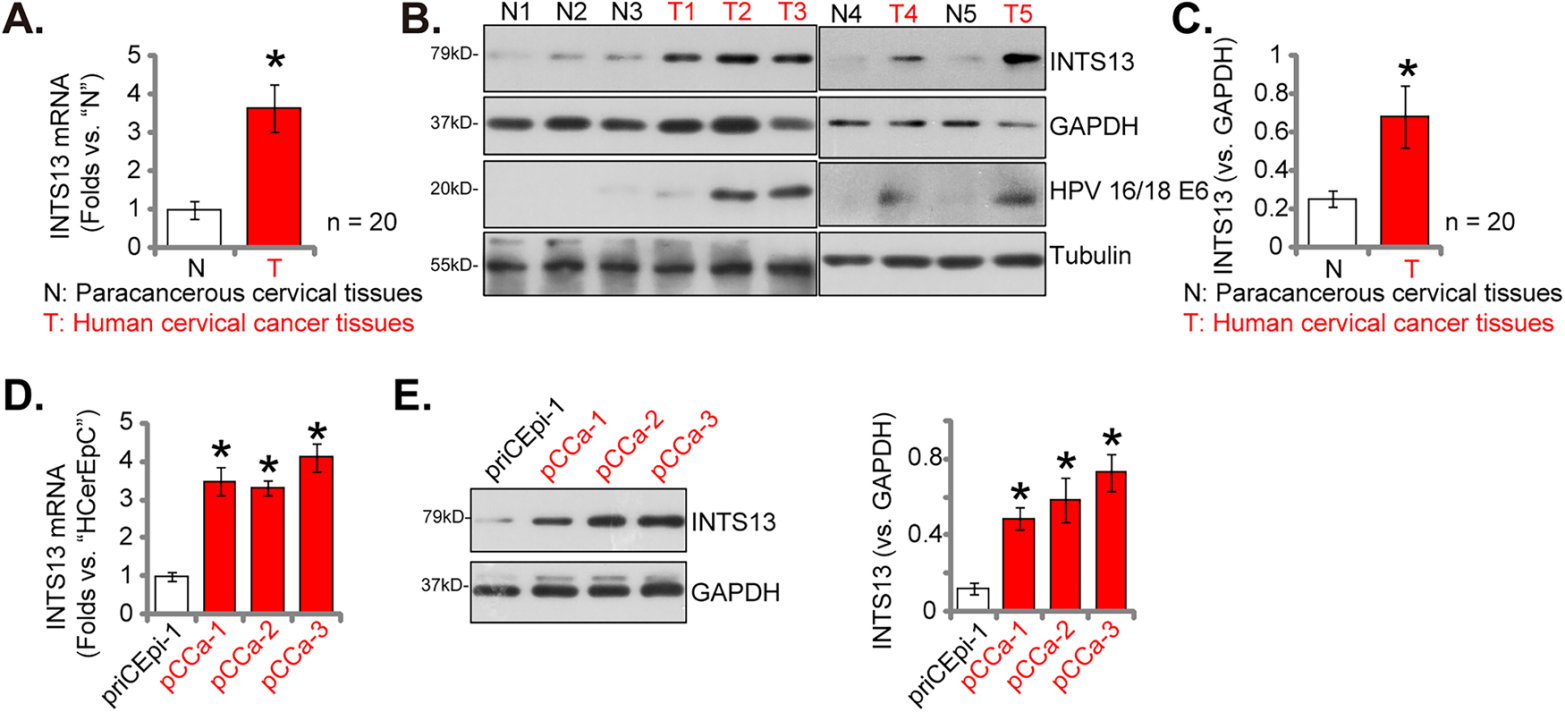
Elevated INTS13 expression in surgically-treated cervical cancer tissues and various cervical cancer cell types. *INTS13* mRNA expression in human cervical cancer tissues (“T”) compared to adjacent paracancerous cervical tissues (“N”) (*n* = 20) (**A**). Representative Western blot showing INTS13 and HPV 16/18 E6 protein levels in five pairs of cervical cancer tissues (“T1”–“T5”) and paracancerous cervical tissues (“N1”–“N5”) (**B**). Quantitative densitometric analysis of INTS13 protein expression in all twenty pairs of cervical cancer and peritumoral tissues (**C**). *INTS13* mRNA and protein levels, respectively, in primary cervical cancer cells (pCCa-1, pCCa-2, pCCa-3) compared to primary human cervical epithelial cells (priCEpi-1) (**D**, **E**). Data are presented as mean ± standard deviation (SD) with *n* = 5 replicates. Asterisks (*) indicate statistically significant differences (*P* < 0.05) compared to the “N” tissues or priCEpi-1 cells. The experiments were independently replicated five times with consistent results.

To validate the expression profile of INTS13 in cervical cancer, we next comprehensively assessed *INTS13* mRNA and protein levels across human cervical tissues and derived cell types. As depicted in Fig. 3A, a statistically significant upregulation of *INTS13* mRNA expression was observed in surgically-treated human cervical cancer tissues (“T”, as reported previously [25]) when compared to adjacent paracancerous cervical tissues (“N”) (*n* = 20). This finding was corroborated by Western blot analysis (Fig. 3B), which demonstrated a significant increase in INTS13 protein levels in cervical cancer tissues (“T1”–“T5”) of five representative patients relative to their peritumoral tissues (“N1”–“N5”) (Fig. 3B). Quantitative densitometric analysis of the Western blot data of all twenty pairs of tissues further confirmed a significant elevation in INTS13 protein expression within human cervical cancer tissues compared to paracancerous cervical tissues (Fig. 3C).

We also assessed the expression of the high-risk HPV oncoprotein E6 (specifically HPV 16/18 E6) in both cervical cancer tissues and their adjacent paracancerous counterparts. As shown in Fig. 3B, Western blot analysis of representative tissue samples clearly demonstrates robust E6 protein expression in the cervical cancer tissues (T2, T3, T4, T5). It was almost undetectable in the adjacent paracancerous cervical tissues (N1, N2, N3, N4, N5) (Fig. 3B).

Furthermore, our investigation extended to INTS13 expression in primary human cervical epithelial cells (priCEpi-1, as reported previously [25]) and primary cervical cancer cells isolated from three individual patients (namely pCCa-1, pCCa-2, pCCa-3, also reported previously [25]). Consistently, both mRNA and protein levels of INTS13 were found to be significantly elevated in all three primary cervical cancer cell types, in stark contrast to the low expression observed in priCEpi-1 (Fig. 3D, E, respectively). Collectively, these findings unequivocally demonstrate a significant increase in INTS13 expression in cervical cancer tissues from surgically-treated patients and in primary cervical cancer cells, while exhibiting low expression in paracancerous tissues and primary human cervical epithelial cells.

### Targeted silencing of INTS13 attenuates malignant phenotypes in cervical cancer cells

**Fig. 4:**
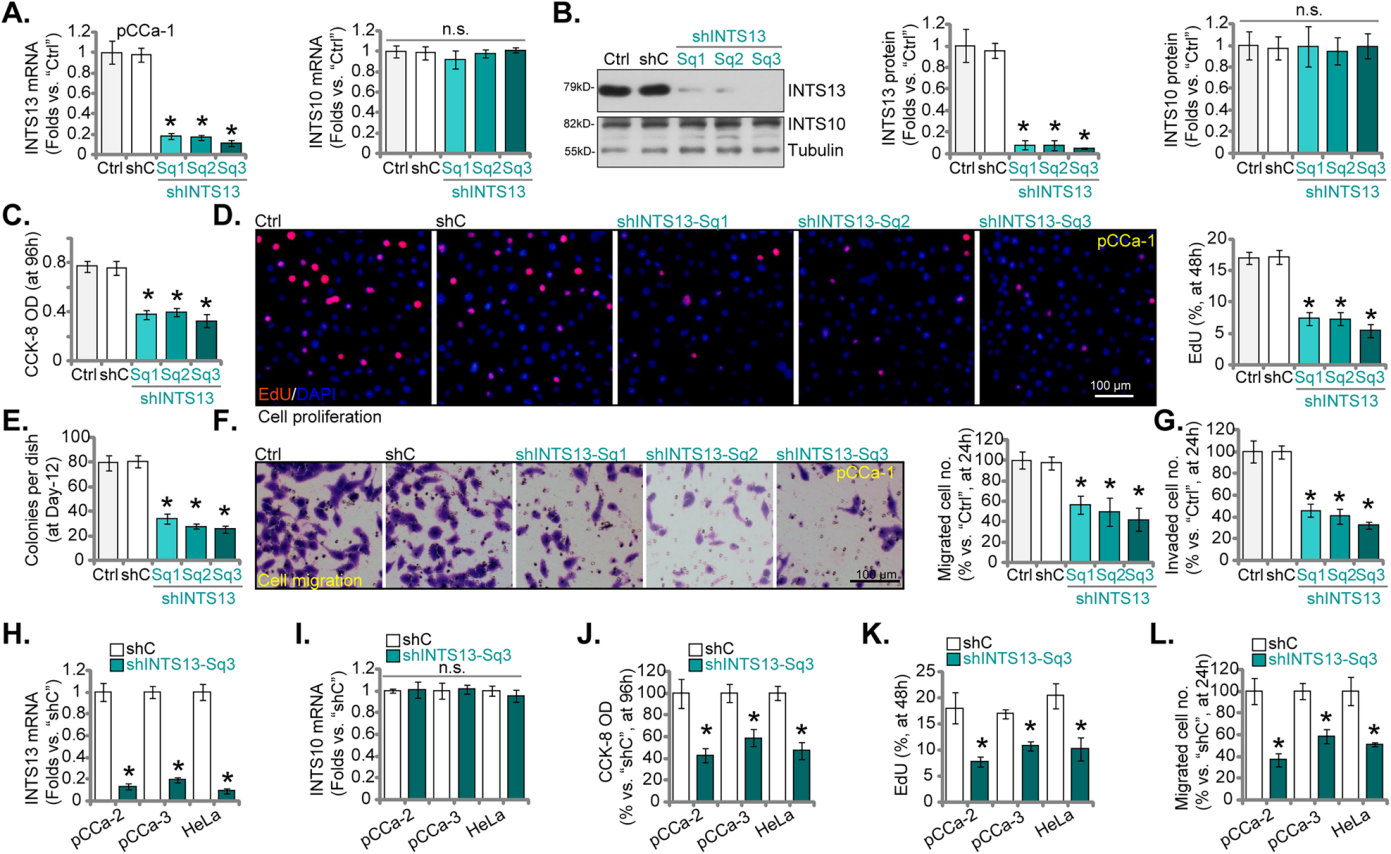
Targeted silencing of INTS13 attenuates malignant phenotypes in cervical cancer cells. Primary pCCa-1 cervical cancer cells were subjected to transfection with three distinct shRNAs (shINTS13-Sq1, shINTS13-Sq2, and shINTS13-Sq3) or control shRNA (shC). Subsequently, the expression levels of *INTS13* mRNA transcript (**A**) and protein (**B**) were assessed, with *INTS10* serving as a control gene. Cells were further cultivated for the indicated time periods, cell viability was determined through CCK-8 (**C**) assay, with cell proliferation assessed via nuclear EdU incorporation (**D**) assay. The clonogenic potential of pCCa-1 cells following INTS13 knockdown was quantified using a colony formation assay (**E**). Transwell assays were performed to evaluate the migratory (**F**) and invasive (**G**) capacities of pCCa-1 cells. Next, shINTS13-Sq3 was utilized to reduce *INTS13* mRNA levels (**H**, **I**) in additional primary cervical cancer cell types (pCCa-2, pCCa-3) and the established HeLa cervical cancer cell line, concurrently assessing *INTS10* mRNA levels. The effect of shINTS13-Sq3-mediated inhibition of INTS13 on the viability and proliferation of pCCa-2, pCCa-3, and HeLa cells was evaluated; specifically, cell viability was assessed by CCK-8 (**J**) assay and cell proliferation by EdU incorporation (**K**) assay. Furthermore, the migratory capacity (**L**) of pCCa-2, pCCa-3, and HeLa cells was determined using the Transwell assay following INTS13 silencing. “Ctrl” stands for the parental control cells. Data are presented as mean ± standard deviation (SD) with *n* = 5 biological replicates. Asterisks (*) indicate statistically significant differences (*P* < 0.05) compared to shC group. “n.s.” denotes no statistically significant difference (*P* > 0.05). The experiments were independently replicated five times with consistent results. Scale bar represents 100 μm.

To ascertain the functional significance of INTS13 to cervical cancer progression, we utilize shRNAs for the targeted suppression of INTS13 expression in primary pCCa-1 SCC cervical cancer cells. As presented in Fig. 4A, three distinct shRNAs (shINTS13-Sq1, shINTS13-Sq2, and shINTS13-Sq3, with non-overlapping sequences) markedly diminished *INTS13* mRNA transcript levels in pCCa-1 primary cells when compared to parental control (Ctrl) or non-targeting shRNA (shC) groups, notably without discernible impact on INTS10 (the control gene) mRNA levels (Fig. 4A). Concomitantly, a significant reduction in INTS13 protein levels was observed with these shRNAs (Fig. 4B), while INTS10 levels remained unperturbed (Fig. 4B). The ramifications of INTS13 silencing on cellular function were first evaluated through CCK-8 cell viability and EdU incorporation proliferation assays. Fig. 4C, D unequivocally demonstrated that the suppression of INTS13 via all three shRNAs led to a significant attenuation of cell viability and proliferation in pCCa-1 cells compared to control groups. Complementary to these findings, the colony formation assay illustrated a substantial decrease in the clonogenic potential of pCCa-1 cells following INTS13 knockdown (Fig. 4E). To ascertain the influence of INTS13 silencing on cellular migratory and invasive capabilities, Transwell assays were conducted. As evidenced in Fig. 4F, the targeted knockdown of INTS13 by shINTS13-Sq1, shINTS13-Sq2, and shINTS13-Sq3 markedly impaired the migratory capacity of pCCa-1 primary cancer cells. Concurrently, the invasive potential of pCCa-1 cells was significantly suppressed following INTS13 silencing (see quantified results in Fig. 4G). These findings underscore a pivotal role for INTS13 in the orchestration of proliferation, clonogenicity, migration, and invasion in pCCa-1 primary cervical cancer cells.

To further substantiate these observations, our investigation was expanded to encompass additional primary cervical cancer cell types (pCCa-2, pCCa-3, as previously reported [25]) alongside the established HeLa cervical cancer cell line. For these experiments, shINTS13-Sq3, previously validated for its potent silencing efficacy in pCCa-1 cells (see Fig. 4A, B), was employed. As depicted in Fig. 4H, I, shINTS13-Sq3 efficaciously decreased *INTS13* mRNA levels in pCCa-2, pCCa-3, and HeLa cells without perturbing *INTS10* mRNA levels. Mirroring the outcomes observed in pCCa-1 cells, the targeted suppression of INTS13 by shINTS13-Sq3 resulted in a significant inhibition of cell viability and proliferation across pCCa-2, pCCa-3, and HeLa cells, as evidenced by CCK-8 and EdU incorporation assays (Fig. 4J, K, respectively). Furthermore, the migratory capacity of pCCa-2, pCCa-3, and HeLa cells was significantly attenuated following INTS13 silencing by shINTS13-Sq3 (Fig. 4L). The consistent manifestation of these inhibitory effects across diverse cervical cancer cell types emphatically underscores the indispensable role of INTS13 in fostering the malignant phenotypic characteristics of cervical cancer cells.

### Targeted silencing of INTS13 induces apoptosis in cervical cancer cells while sparing cervical epithelial cells

**Fig. 5:**
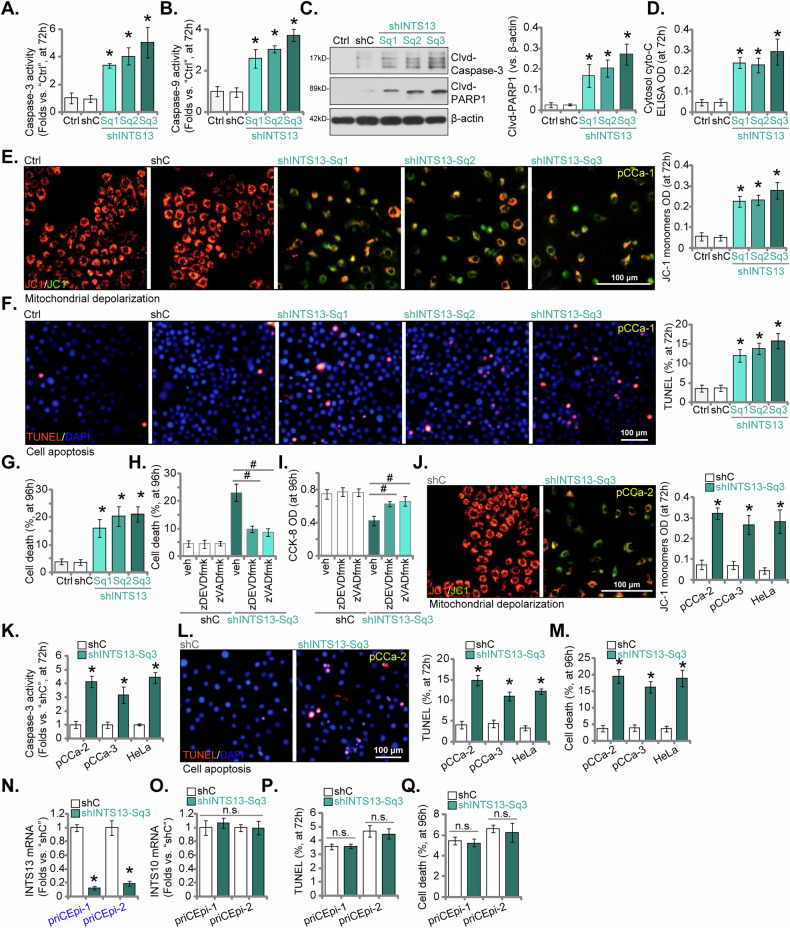
Targeted silencing of INTS13 induces apoptosis in cervical cancer cells while sparing cervical epithelial cells. Primary pCCa-1 cervical cancer cells were transfected with three distinct shRNAs (shINTS13-Sq1, shINTS13-Sq2, and shINTS13-Sq3) or control shRNAs (shC). Cells were further cultivated for the indicated time periods, subsequently, the enzymatic activities of Caspase-3 (**A**) and Caspase-9 (**B**) were assessed. Western blot analysis (**C**) was performed to examine the proteolytic cleavage of PARP and Caspase-3 (Cld-PARP1 and Cld-Caspase-3, respectively). Cytosol cytochrome C levels were also measured using an ELISA kit (**D**). Mitochondrial depolarization was assessed using JC-1 staining to determine the green monomers intensity (**E**). The nuclear TUNEL staining assay (**F**) and Trypan blue staining assay (**G**) were employed to assess apoptotic cells and overall cell death. The efficacy of the pan-caspase inhibitor z-VAD-fmk (50 μM) and the caspase-3 inhibitor z-DEVD-fmk (50 μM) in inhibiting INTS13 silencing (shINTS13-Sq3)-induced cell death (**H**) and viability (CCK-8 OD) reduction (**I**) was evaluated. The apoptotic effects of INTS13 silencing (shINTS13-Sq3) were examined in additional primary cervical cancer cell types (pCCa-2, pCCa-3) and the established HeLa cervical cancer cell line, assessing mitochondrial depolarization (**J**), Caspase-3 activity (**K**), prevalence of TUNEL-positive cells (**L**), and cellular death (**M**). The effect of shINTS13-Sq3 was investigated in non-cancerous primary human cervical epithelial cells (priCEpi-1 and priCEpi-2). *INTS13* mRNA silencing (**N**) and *INTS10* mRNA levels (**O**) were assessed in these cervical epithelial cells. Apoptosis induction in priCEpi-1 and priCEpi-2 cells was evaluated by TUNEL staining (**P**), and cell death was tested via Trypan blue staining assays (**Q**). “Ctrl” stands for the parental control cells. Data are presented as mean ± standard deviation (SD) with *n* = 5 biological replicates. Asterisks (*) indicate statistically significant differences (*P* < 0.05) compared to shC group. # *P* < 0.05 (**H**, **I**). “n.s.” denotes no statistically significant difference (*P* > 0.05). The experiments were independently replicated five times with consistent results. Scale bar represents 100 μm.

To comprehensively delineate the functional ramifications of INTS13 inhibition, we investigated its impact on the induction of apoptosis within cervical cancer cells. As shown, silencing of INTS13 in primary pCCa-1 cervical cancer cells, achieved through the application of three distinct shRNAs (shINTS13-Sq1, shINTS13-Sq2, and shINTS13-Sq3, see Fig. 4), elicited a statistically significant augmentation in the enzymatic activities of both Caspase-3 (Fig. 5A) and Caspase-9 (Fig. 5B), relative to parental control (Ctrl) or non-targeting shRNA (shC) groups. This activation of the intrinsic apoptotic cascade was further corroborated by Western blot analysis, which demonstrated an increased proteolytic cleavage of PARP and Caspase-3 (manifested as Cld-PARP1 and Cld-Caspase-3, respectively) subsequent to INTS13 silencing (Fig. 5C). In addition, the levels of cytosol cytochrome C levels were increased in INTS13-silenced pCCa-1 cells (Fig. 5D). Mitochondrial depolarization, an early event characteristic of the apoptotic process, was assessed utilizing JC-1 staining. INTS13 silencing precipitated a conspicuous decline in the red-to-green fluorescence ratio, serving as an indicator of compromised mitochondrial membrane potential within pCCa-1 cells (Fig. 5E). Furthermore, the nuclear TUNEL staining assay revealed a significant increase in the population of apoptotic cells subsequent to INTS13 silencing (Fig. 5F), and Trypan blue staining assay independently verified death within pCCa-1 cells upon INTS13 suppression (Fig. 5G). The pan-caspase inhibitor z-VAD-fmk and the caspase-3 inhibitor z-DEVD-fmk effectively inhibited the INTS13 silencing (shINTS13-Sq3)-induced overall cell death and viability (CCK-8 OD) reduction (Fig. 5H, I, respectively) within pCCa-1 cells.

To broaden the generalizability of these findings, we extended our investigation to examine the apoptotic effects of INTS13 silencing (specifically employing shINTS13-Sq3) in additional primary cervical cancer cell types (pCCa-2, pCCa-3) and the established HeLa cervical cancer cell line. As shown, shINTS13-Sq3 significantly induced mitochondrial depolarization (Fig. 5J), augmented Caspase-3 activity (Fig. 5K), increased the prevalence of TUNEL-positive cells (Fig. 5L), and robustly promoted cellular death (Fig. 5M) across pCCa-2, pCCa-3, and HeLa cells. Crucially, to ascertain the selective specificity of INTS13’s pro-apoptotic role within malignant cellular contexts, we investigated the effect of shINTS13-Sq3 in non-cancerous primary human cervical epithelial cells (priCEpi-1 and priCEpi-2, as reported previously [25]). As demonstrated in Fig. 5N, O, shINTS13-Sq3 effectively achieved *INTS13* mRNA silencing in these epithelial cells without eliciting any detectable alteration in *INTS10* mRNA levels. However, in stark contrast to the observations in cervical cancer cells, INTS13 silencing in priCEpi-1 and priCEpi-2 cells failed to induce apoptosis, as evidenced by unchanged TUNEL staining (Fig. 5P) and the absence of a statistically significant increase in cell death (Fig. 5Q). These findings underscore that INTS13 silencing selectively elicits apoptosis in cervical cancer cells.

### Genetic ablation of INTS13 attenuates malignant phenotypes and induces apoptosis in primary cervical cancer cells

**Fig. 6:**
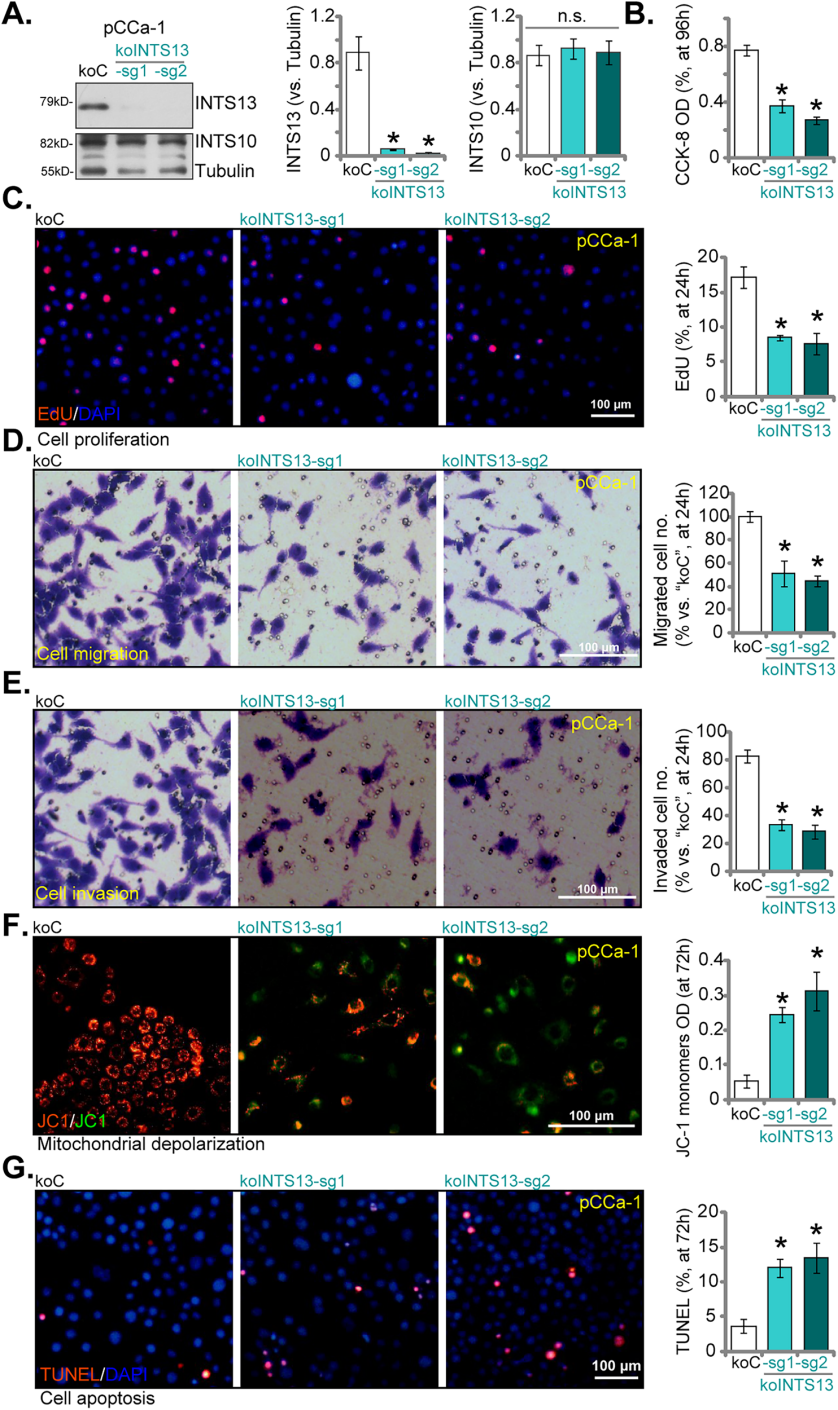
Genetic ablation of INTS13 attenuates malignant phenotypes and induces apoptosis in primary cervical cancer cells. Primary pCCa-1 human cervical cancer cells were established via lentiviral CRISPR/Cas9 transfection, incorporating Cas9-expressing construct plus either control sgRNA (“koC”) or INTS13-specific sgRNAs (koINTS13-sg1/koINTS13-sg2). Expression levels of relevant proteins were subsequently analyzed (**A**). These cells were then cultured under specified conditions to assess malignant phenotypes: cell viability (CCK-8 assay, **B**), cell proliferation (EdU-positive nuclei ratio, **C**), and in vitro cell migration and invasion (Transwell assays, **D**, **E**). Additionally, mitochondrial membrane potential was determined by JC-1 staining (**F**), and cell apoptosis by nuclear TUNEL staining (**G**). Data are presented as mean ± standard deviation (SD) with *n* = 5 biological replicates. Asterisks (*) indicate statistically significant differences (*P* < 0.05) compared to “koC” group. “n.s.” denotes no statistically significant difference (*P* > 0.05). The experiments were independently replicated five times with consistent results. Scale bar represents 100 μm.

To further substantiate the pivotal role of INTS13 in the progression of cervical cancer, we employed CRISPR/Cas9 gene editing technology to generate INTS13 knockout (koINTS13) primary pCCa-1 cervical cancer cell lines. This was achieved using two distinct single guide RNAs (sgRNAs), designated sg1 and sg2. Both the koINTS13-sg1 and koINTS13-sg2 caused a profound reduction in INTS13 protein levels when compared to the control knockout (koC) (Fig. 6A), while notably, INTS10 protein levels remained unaltered (Fig. 6A). The consequential impact of INTS13 knockout on cell viability and proliferation was assessed through the application of CCK-8 and EdU incorporation assays, respectively. Figure 6B, C demonstrated that the genetic ablation of INTS13 significantly impeded the viability and proliferative capacity of pCCa-1 cells relative to koC cells. Furthermore, we extended our investigation to evaluate the effect of INTS13 knockout on cell migration and invasion, utilizing Transwell assays. As presented in Fig. 6D, E, both koINTS13-sg1 and koINTS13-sg2 cells displayed a markedly diminished migratory and invasive capability when compared to koC cells. Mitochondrial depolarization was again assessed using JC-1 staining. As illustrated in Fig. 6F, the genetic knockout of INTS13 led to a pronounced decrease in the red-to-green fluorescence ratio, signifying a critical loss of mitochondrial membrane potential within pCCa-1 cells. The TUNEL assay provided additional corroboration, revealing a significant increase in apoptotic cells following INTS13 knockout (Fig. 6G). These collective data definitively confirm that the genetic ablation of INTS13 inhibited malignant phenotypes and induced apoptosis in primary pCCa-1 cervical cancer cells.

### Overexpression of INTS13 enhances malignant phenotypes in cervical cancer cells

**Fig. 7:**
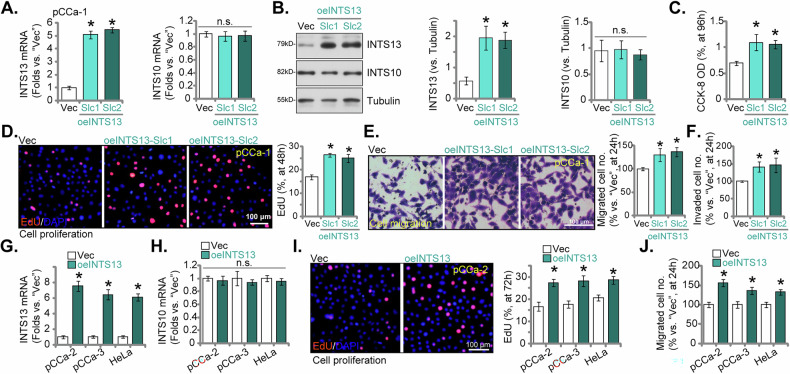
Overexpression of INTS13 enhances malignant phenotypes in cervical cancer cells. The primary pCCa-1 cervical cancer cells were engineered to stably overexpress INTS13 using a lentiviral construct (oeINTS13). Two independently derived stable cell selections, oeINTS13-Slc1 and oeINTS13-Slc2, were assessed for elevated *INTS13* mRNA transcript levels and unaffected *INTS10* mRNA expression compared to vector control (Vec) cells (**A**). Western blot analysis confirmed increased INTS13 protein levels in both oeINTS13-Slc1 and oeINTS13-Slc2 cells, with INTS10 protein expression tested as a control (**B**). Cells were further cultivated for designated time, cellular viably and proliferation were assessed through CCK-8 (**C**) and nuclear EdU incorporation (**D**) assays, respectively. Furthermore, the migratory (**E**) and invasive (**F**) capabilities were tested. The experiments expanded to other primary cervical cancer cell types (pCCa-2, pCCa-3) and the established HeLa cell line, utilizing the same lentiviral INTS13 overexpression construct (oeINTS13). *INTS13* (**G**) and *INTS10* mRNA transcripts (**H**) were assessed in these cells following overexpression. Cells were further cultivated for designated hours, the augmentation of proliferation (**I**) and migratory ability (**J**) in pCCa-2, pCCa-3, and HeLa cells upon INTS13 overexpression was assessed via nuclear EdU incorporation and Transwell assays, respectively. Data are presented as mean ± standard deviation (SD) with *n* = 5 biological replicates. Asterisks (*) indicate statistically significant differences (*P* < 0.05) compared to the Vec group. “n.s.” denotes no statistically significant difference (*P* > 0.05). The experiments were independently replicated five times with consistent results. Scale bar represents 100 μm.

To further elucidate the pro-oncogenic role of INTS13, we engineered primary pCCa-1 cervical cancer cells to stably overexpress INTS13 utilizing a lentiviral construct. As presented in Fig. 7A, two independently derived stable cell selections, designated oeINTS13-Slc1 and oeINTS13-Slc2, exhibited significantly elevated *INTS13* mRNA transcript levels when compared to vector control (Vec) cells, without concurrently affecting *INTS10* mRNA expression (Fig. 7A). Western blot analysis provided further confirmation, revealing a substantial increase in INTS13 (but not INTS10) protein levels in both oeINTS13-Slc1 and oeINTS13-Slc2 cells (Fig. 7B). Figure 7C, D demonstrated that the augmented expression of INTS13 significantly enhanced the viability (CCK-8 OD) and proliferative capacity (nuclear EdU incorporation) of pCCa-1 cells relative to Vec cells. Furthermore, both oeINTS13-Slc1 and oeINTS13-Slc2 pCCa-1 cells displayed significantly enhanced migratory capabilities when compared to Vec pCCa-1 cells (Fig. 7E). Similarly, the invasive potential of pCCa-1 cells was demonstrably increased upon INTS13 overexpression (Fig. 7F). Next, our investigation was expanded to encompass other primary cervical cancer cell types (pCCa-2, pCCa-3) and the established HeLa cell line, utilizing the same lentiviral INTS13 overexpression construct. As depicted in Fig. 7G, *INTS13* mRNA levels were significantly increased in pCCa-2, pCCa-3, and HeLa cells following overexpression (oeINTS13), while *INTS10* mRNA transcripts remained unaffected (Fig. 7H). Consistent with the observations in pCCa-1 cells, INTS13 overexpression significantly augmented the proliferation of pCCa-2, pCCa-3, and HeLa cells, as evidenced by EdU incorporation assays (Fig. 7I). Moreover, the migratory ability of these cervical cancer cells was significantly enhanced upon INTS13 overexpression (Fig. 7J). These consistent findings across multiple cervical cancer cell types underscore the pervasive role of INTS13 in promoting the malignant phenotypes associated with cervical cancer.

### hnRNPC mediates INTS13-induced malignant phenotypes in cervical cancer cells

**Fig. 8:**
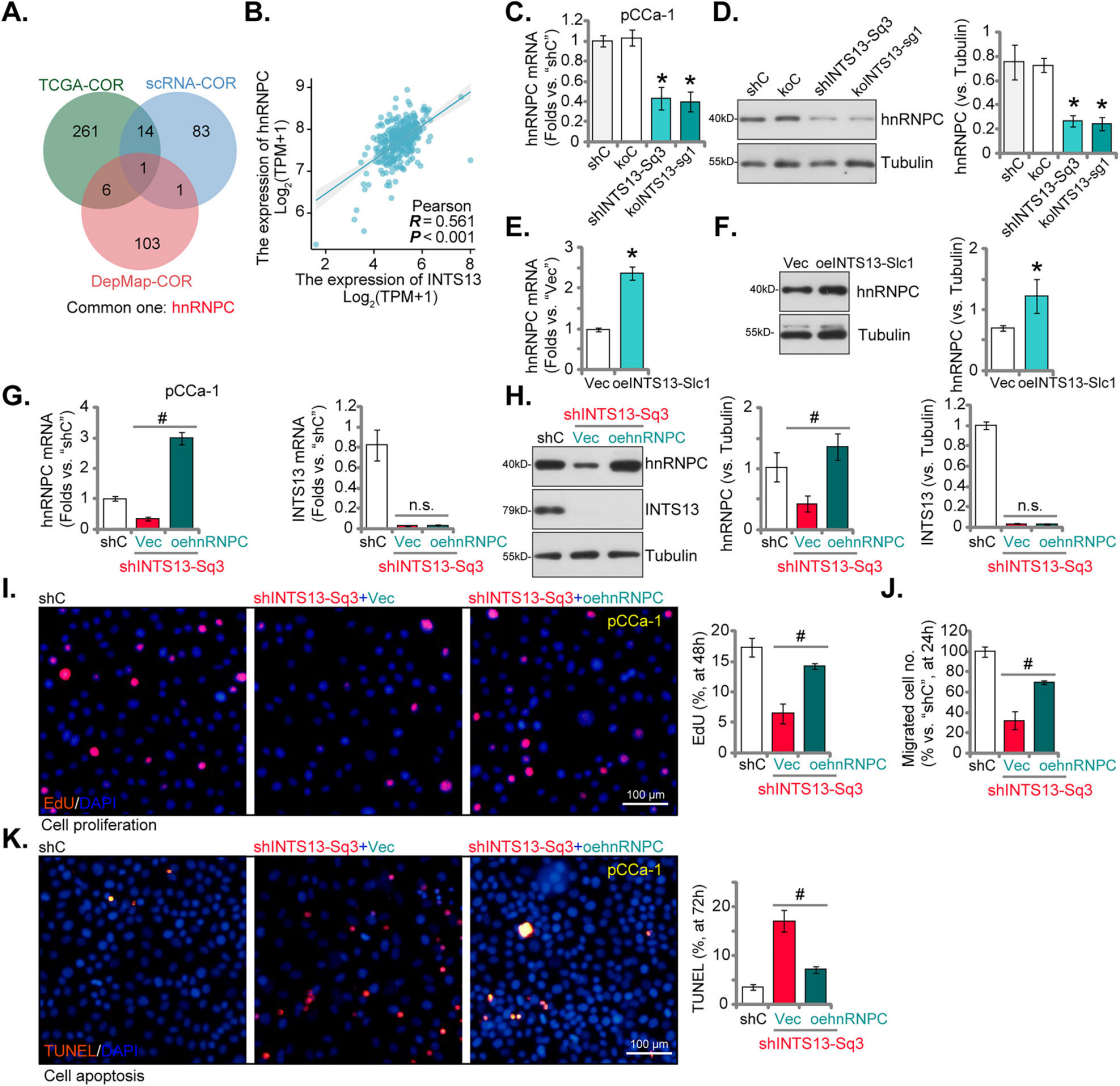
hnRNPC mediates INTS13-induced malignant phenotypes in cervical cancer cells. The Venn diagram illustrates the intersection of the top 100 co-expressed genes (TCGA transcriptome), top 100 co-expressed proteins (DepMap proteome), and top 100 co-expressed genes identified from single-cell sequencing’s malignant epithelial cell subpopulations, collectively pinpointing hnRNPC as the singular common molecular entity (**A**). The scatter plot was then generated from the TCGA data to investigate the correlation between the expression levels of *hnRNPC* and *INTS13* (**B**). Primary pCCa-1 cervical cancer cells were subjected to targeted silencing (using shINTS13-Sq3) or genetic knockout (by koINTS13-sg1) of INTS13, followed by quantitative RT-PCR (**C**) and Western blot analysis (**D**) to assess hnRNPC expression at both the mRNA and protein levels. INTS13 was ectopically overexpressed (oeINTS13-Slc1) in pCCa-1 cells, and quantitative RT-PCR (**E**) and Western blot analysis (**F**) were performed to determine the resulting changes in *hnRNPC* mRNA and protein levels. A lentiviral hnRNPC-overexpressing construct was introduced into INTS13-silenced pCCa-1 cells to restore hnRNPC expression, with confirmation via quantitative RT-PCR (**G**) and Western blot analysis (**H**). Cells were further cultivated for the indicated time periods, and an EdU incorporation assay was conducted to evaluate cellular proliferation capacity after hnRNPC restoration (**I**), with cell migration tested via Transwell assay (**J**), and cell apoptosis tested via TUNEL staining assay (**K**). Data are presented as mean ± standard deviation (SD) with *n* = 5 biological replicates. Asterisks (*) indicate statistically significant differences (*P* < 0.05) compared to the “shC”/“Vec” treatment. ^#^ indicates *P* < 0.05 (**G**–**K**). “n.s.” denotes no statistically significant difference (*P* > 0.05). The experiments were independently replicated five times with consistent results. Scale bar represents 100 μm.

To identify potential downstream effectors responsible for mediating the oncogenic functions of INTS13, we conducted a comprehensive bioinformatics analysis. This involved performing a correlation analysis utilizing both TCGA cervical cancer transcriptome data and DepMap proteome data. Subsequently, the top 100 co-expressed genes (TCGA, see Fig. 1) and proteins (DepMap) derived from these independent datasets were intersected with the top 100 co-expressed genes identified from single-cell cancer cell subpopulations (see Fig. 2). This intersection analysis unequivocally pinpointed hnRNPC (heterogeneous nuclear ribonucleoprotein C) as the singular common molecular entity (Fig. 8A). A subsequent scatter plot generated from the TCGA data further elucidated a robust positive correlation between the expression levels of hnRNPC and INTS13 (Fig. 8B). To experimentally validate this observed correlative relationship, we investigated the impact of INTS13 modulation on hnRNPC expression within primary pCCa-1 cancer cells. As demonstrated in Fig. 8C, D, both the targeted silencing (using shINTS13-Sq3, see Figs. 4 and 5) and genetic knockout (by koINTS13-sg1, see Fig. 5) of INTS13 significantly attenuated the expression of hnRNPC at both the mRNA and protein levels, respectively. Conversely, the ectopic overexpression of INTS13 (oeINTS13-Slc1, see Fig. 7) in pCCa-1 cells resulted in a marked elevation in both *hnRNPC* mRNA and protein levels (Fig. 8E, F), thereby confirming that INTS13 positively regulates hnRNPC expression in pCCa-1 cervical cancer cells.

To ascertain whether hnRNPC serves as a critical downstream mediator of INTS13’s pro-oncogenic effects, we carried targeted rescue experiments. Specifically, we restored hnRNPC expression in INTS13-silenced pCCa-1 cells through the introduction of a lentiviral hnRNPC-overexpressing construct (“+oehnRNPC”, Fig. 8G, H). The re-establishment of hnRNPC expression significantly ameliorated the inhibitory effect of INTS13 silencing (by shINTS13-Sq3) on cellular proliferation, as evidenced by EdU incorporation (Fig. 8I). Similarly, the impaired migratory capacity induced by INTS13 silencing was inhibited upon hnRNPC restoration (Fig. 8J). Furthermore, the augmented apoptosis observed subsequent to INTS13 silencing was significantly attenuated when hnRNPC expression was reinstated (Fig. 8K). These collective and compelling findings establish hnRNPC as an indispensable downstream mediator through which INTS13 exerts its pro-cancerous effects within cervical cancer cells.

### The transcription factor ZNF384 governs INTS13 expression in cervical cancer cells

**Fig. 9:**
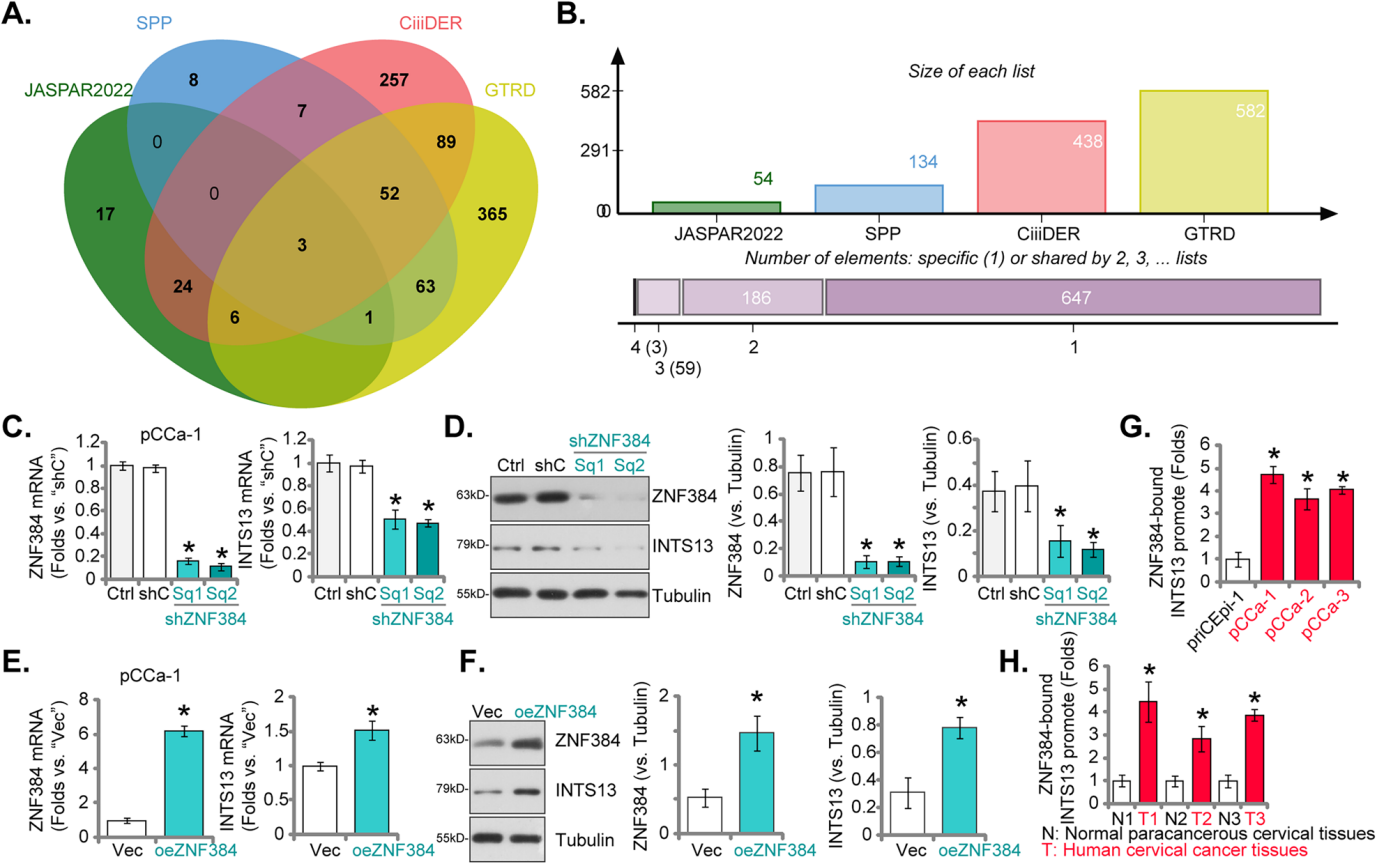
The transcription factor ZNF384 governs INTS13 expression in cervical cancer cells. A comprehensive bioinformatics analysis was performed to predict transcription factors capable of binding to the *INTS13* promoter region (defined as 2000 bp upstream to 100 bp downstream of the transcription start site, TSS), leveraging four distinct databases—UCSC-JASPAR2022, GTRD, SPP, and CiiiDER—to identify common upstream transcription factors (**A**, **B**). shRNA-mediated silencing of ZNF384 was achieved through the application of two distinct lentivirus-packed shRNAs (Sq1 and Sq2), followed by assessment of *ZNF384*-*INTS13* mRNA (**C**) and protein (**D**) expression in pCCa-1 cells. Conversely, ZNF384 was ectopically overexpressed in pCCa-1 cells, after which *ZNF384*-*INTS13* mRNA (**E**) and protein (**F**) levels were evaluated. The chromatin immunoprecipitation (ChIP) assays were conducted to detect the direct physical association of ZNF384 with the *INTS13* promoter in primary cervical cancer cells (pCCa-1, pCCa-2, pCCa-3) compared to human cervical epithelial cells (priCEpi-1) (**G**). Furthermore, ChIP assays were performed on clinical cervical cancer tissues (T1, T2, T3) in contrast to adjacent paracancerous cervical tissues (N1, N2, N3) (**H**). Data are presented as mean ± standard deviation (SD) with *n* = 5 biological replicates. Asterisks (*) indicate statistically significant differences (*P* < 0.05) compared to the “shC”/“Vec” treatment or “N” tissues or priCEpi-1 cells. The experiments were independently replicated five times with consistent results.

Since both mRNA and protein expression levels were upregulated in cervical cancer tissues and cells (see Figs. 1–3), we next elucidate the transcriptional mechanisms governing INTS13 expression and performed a comprehensive bioinformatics analysis to predict transcription factors capable of binding to the *INTS13* promoter region (defined as 2000 bp upstream to 100 bp downstream of the transcription start site, TSS). Leveraging four distinct databases—UCSC-JASPAR2022, GTRD, SPP, and CiiiDER—our analysis converged on the identification of three common upstream transcription factors: YY1, SPI1, and ZNF384 (Fig. 9A, B). Notably, ZNF384 consistently exhibited the highest prediction score among these candidates, strongly implicating its preeminent regulatory role. To experimentally validate the predicted regulatory interplay between ZNF384 and INTS13, we precisely modulated ZNF384 expression in cervical cancer cells. As depicted in Fig. 9C, D, shRNA-mediated silencing of ZNF384, achieved through the application of two distinct shRNAs (Sq1 and Sq2), resulted in a significant concomitant downregulation of both *INTS13* mRNA (Fig. 9C) and protein (Fig. 9D) expression in pCCa-1 cells. Contrarily, shRNA-induced silencing of two other predicted transcription factors, YY1 and SPI1, failed to significantly alter INTS13 expression in pCCa-1 cells (Data not shown). The ectopic overexpression of ZNF384 in pCCa-1 cells precipitated a marked upregulation of *INTS13* mRNA and protein levels (Fig. 9E, F), thereby conclusively confirming that ZNF384 positively regulates INTS13 expression in pCCa-1 cells. To definitively establish the direct physical association of ZNF384 with the *INTS13* promoter, we conducted chromatin immunoprecipitation (ChIP) assays. The results unequivocally demonstrated a substantially increased binding affinity of ZNF384 to the predicted *INTS13* promoter region in primary cervical cancer cells (pCCa-1, pCCa-2, pCCa-3) when compared to human cervical epithelial cells (priCEpi-1) (Fig. 9G). Furthermore, ChIP assays performed on clinical cervical cancer tissues (T1, T2 and T3, see Fig. 3) revealed significantly enhanced ZNF384 occupancy at the *INTS13* promoter locus in contrast to adjacent paracancerous cervical tissues (N1, N2, and N3) (Fig. 9H). These findings collectively establish ZNF384 as a pivotal transcription factor that directly associates with and positively governs INTS13 expression in the context of cervical cancer.

### Targeted silencing of INTS13 impedes cervical cancer xenograft tumor growth in nude mice

**Fig. 10:**
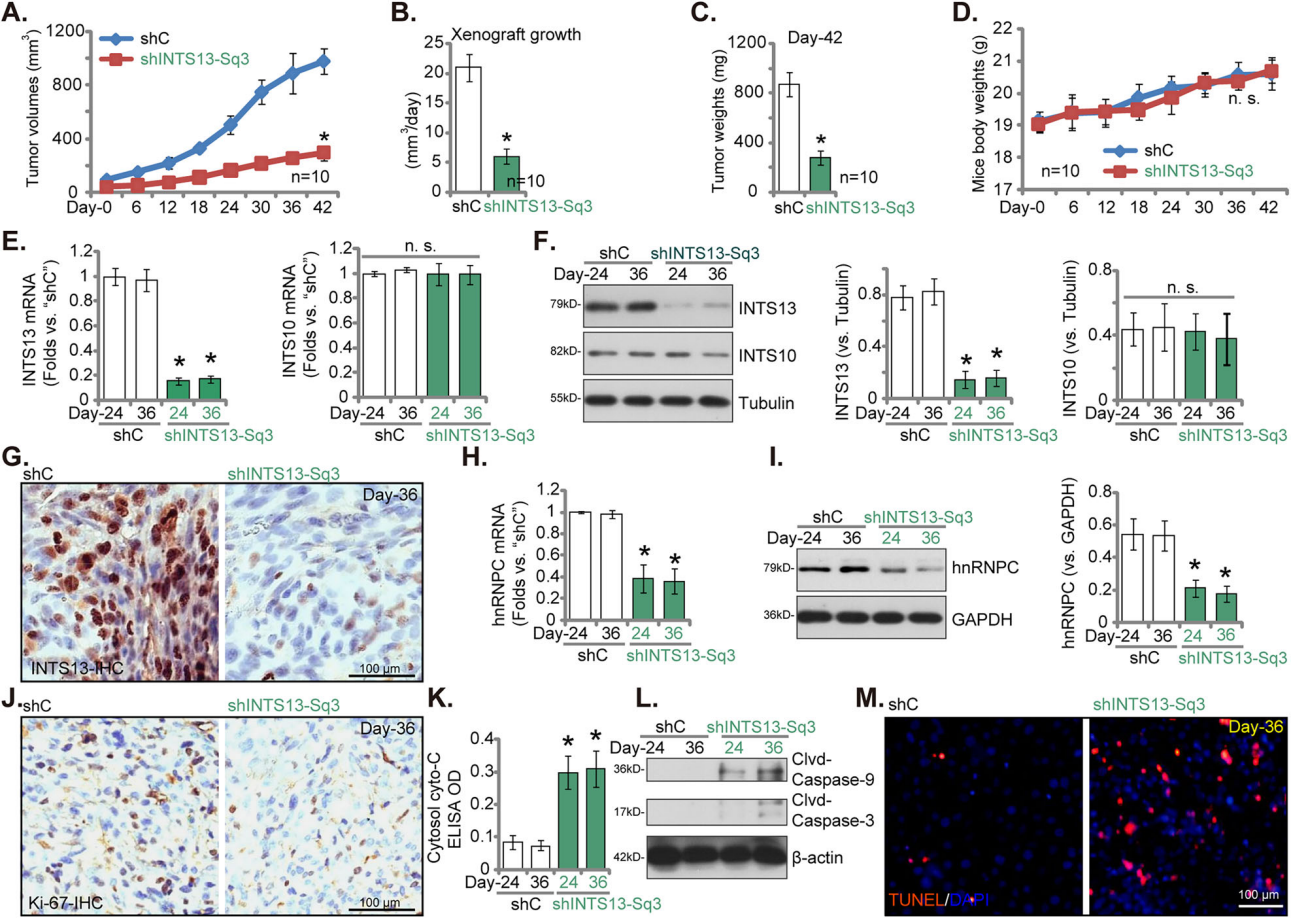
Targeted silencing of INTS13 impedes cervical cancer xenograft tumor growth in nude mice. Primary pCCa-1 cervical cancer cells, stably expressing either a control shRNA (shC) or an INTS13-targeting shRNA (shINTS13-Sq3), were injected to establish xenograft tumors, with tumor growth monitored from Day–0, commencing 21 days post-injection (**A**). The average tumor growth rate was quantitatively assessed (**B**), and final tumor weights were recorded at Day–42 (**C**). Mouse body weights were also consistently monitored (**D**). Upon surgical excision of designated tumor tissues (one xenograft per group, at Day-24 and Day–36, a total of four tumors), analyses were performed to determine the expression profiles of listed proteins and mRNAs (**E**, **F**, **H**, **I**, **L**). Immunohistochemical (IHC) staining was utilized to further confirm the downregulation of INTS13 in the shINTS13-Sq3 tumors (**G**). Furthermore, IHC staining for Ki67, a well-established marker of cellular proliferation, was conducted (**J**). The levels of cytosolic Cytochrome c in the listed tumor lysates were analyzed (**K**). A TUNEL assay was also performed to quantify apoptotic cells within the INTS13-silenced tumor tissues (**M**). Data are presented as mean ± standard deviation (SD). Statistical significance was determined using a *P*-value of less than 0.05 (*P < 0.05) compared to the “shC” group. “n.s.” indicates non-significant differences (*P* > 0.05). For analyses related to tumor volume (**A**), tumor growth rate (**B**), tumor weight (**C**), and mouse body weight (**D**), data were obtained from ten mice per group. For analyses of (**E**–**M**), each xenograft was divided into five segments for individual analysis. Scale bar represents 100 μm.

To evaluate the in vivo oncogenic contribution of INTS13, we established subcutaneous xenograft tumors in nude mice. This was achieved by injecting primary pCCa-1 cervical cancer cells stably expressing either a control shRNA (shC) or an INTS13-targeting shRNA (shINTS13-Sq3). Tumor growth was monitored from Day 0, commencing 21 days post-injection. As depicted in Fig. 10A, xenograft tumors derived from INTS13-silenced pCCa-1 cells (shINTS13-Sq3) exhibited significantly attenuated growth rates and demonstrably smaller tumor volumes when compared to those formed by control shRNA pCCa-1 cells. This pronounced reduction in growth was further quantitatively corroborated by a significant decrease in the average tumor growth rate (Fig. 10B) and reduced tumor weights recorded at Day 42 (Fig. 10C). No discernible impact on mouse body weights was observed (Fig. 10D). Upon surgical excision of the designated tumor tissues (one xenograft per group of each group, at Day-24 and Day-36), we proceeded to analyze the expression profiles of INTS13 and hnRNPC, alongside key markers indicative of cellular proliferation and apoptosis. The INTS13-silenced xenograft tumors consistently displayed a significant reduction in both *INTS13* mRNA and protein expression relative to control shC tumors (Fig. 10E, F), while expression of the control gene INTS10 remained unperturbed (Fig. 10E, F). In addition, IHC staining images further confirmed downregulation of INTS13 in the shINTS13-Sq3 tumors (Fig. 10G). Consistent with our preceding in vitro observations, both hnRNPC mRNA and protein expression were also significantly downregulated within the INTS13-silenced tumors (Fig. 10H, I). Furthermore, IHC staining revealed a marked decrease in Ki67-positive cells within INTS13-silenced tumor tissues compared to control tumors (Fig. 10J), thereby signifying a substantial reduction in tumor cell proliferation in vivo. Concurrently, the analysis of apoptotic markers demonstrated an elevated level of cytosolic cytochrome c (Fig. 10K) and enhanced proteolytic cleavage of both Caspase-9 and Caspase-3 (Fig. 10L) in INTS13-silenced tumors. The TUNEL fluorescence assay in the tumor sections provided additional corroboration, demonstrating a significant increase in apoptotic cells within the INTS13-silenced tumor tissues (Fig. 10M). These in vivo findings collectively support that INTS13 actively promotes cervical cancer growth in vivo, fostering tumor growth and concurrently inhibiting apoptosis.

## Discussion

INTS13 is an indispensable subunit of the Integrator complex, a crucial multi-protein machinery that precisely regulates gene expression by terminating RNA polymerase II transcription in the promoter-proximal region of genes [20–22]. Its molecular mechanism involves functioning as a structural component of the Integrator tail/arm module (INTS10/13/14/15), which exhibits nucleic acid binding affinity and acts as a platform for transcription factor recruitment [20–22]. INTS13 directly interacts with the Integrator’s cleavage module, an interaction critical for efficient snRNA processing [20–22]. The dysregulation of INTS13 and the broader Integrator complex is unequivocally linked to various human diseases, and specific INTS13 variants contribute to recessive developmental ciliopathies [5]. Few studies have focused on the expression, functional role and mechanism of action of INTS13 in human cancer. Wang et al., reported that higher expression of INTS13 correlates with poorer OS in hepatocellular carcinoma (HCC) patients, promoting HCC cell proliferation and inhibiting apoptosis [4]. A pan-cancer study by Federico et al. showed that transcriptional deregulation of *INTS13* is significantly prevalent, occurring in 8 of 22 analyzed cancer types, with significant upregulation observed in rectum AC, small cell lung cancer, and cholangiocarcinoma [31].

Our results provided compelling evidence that INTS13 represents a promising therapeutic target for cervical cancer. Our analysis of TCGA datasets revealed a consistent overexpression of *INTS13* across various histological subtypes of cervical cancer, which significantly correlated with advanced tumor T-stage and predicted poorer OS, particularly in patients with higher body mass index. Furthermore, scRNA sequencing pinpointed INTS13 expression predominantly within malignant epithelial cells of the tumor microenvironment, where its expression was associated with genes involved in critical cellular processes such as RNA processing and mitochondrial functions. In vitro functional studies corroborated these findings, demonstrating that genetic silencing or CRISPR/Cas9-mediated knockout of INTS13 markedly inhibited the proliferation, migration, and invasion of primary cervical cancer cells while selectively inducing apoptosis. Conversely, INTS13 overexpression enhanced these malignant phenotypes. The in vivo animal studies demonstrated that targeted silencing of INTS13 effectively inhibited pCCa-1 cervical cancer xenograft growth in nude mice. This intervention also led to reduced cellular proliferation and increased apoptosis. These robust correlations and functional validations strongly support the notion that INTS13 plays a pro-oncogenic role in cervical cancer.

hnRNPC is an RNA-binding protein critical for nuclear RNA processing. Its core function involves binding to pre-messenger RNA (pre-mRNA), initiating the formation of 40S heterogeneous nuclear ribonucleoprotein (hnRNP) particles [32, 33]. This interaction influences pre-mRNA processing, particularly alternative splicing, and impacts mRNA stability and translation [32, 33]. hnRNPC also functions as an m6A reader, connecting m6A-modified RNAs to splicing regulation [32, 33]. Ultimately, hnRNPC is indispensable for gene expression, and its dysregulation is linked to various pathologies, including cancer [32, 33]. Zhang et al. have shown that hnRNPC is overexpressed in various prevalent malignancies, such as liver and lung cancers, and correlates with unfavorable prognoses and tumor immunity [30]. Suppressing hnRNPC significantly curtailed the proliferation, metastasis, and infiltration of liver cancer cells [30]. Wang et al., have shown that hnRNPC is overexpressed in prostate cancer, correlating with poor clinical outcomes and inhibiting tumor proliferation and metastasis when silenced, suggesting its potential as a prognostic and therapeutic target [34]. Xia et al., reported that hnRNPC is highly expressed in pancreatic cancer and contributes to radiation resistance by activating the RhoA/ROCK2-YAP/TAZ pathway and promoting DNA damage repair [35]. Fischl and colleagues reported that hnRNPC overexpression establishes specific alternative cleavage and polyadenylation (APA) profiles in metastatic colon cancer cells by regulating poly(A) site selection in cancer-implicated genes like MTHFD1L [36].

The expression of hnRNPC is also overexpressed in cervical cancer and significantly contributes to lymphatic metastasis [37]. It achieves this by promoting the production of tumor-related gene variants in an m6A-dependent manner, specifically by regulating the exon skipping of FOXM1 through its m6A-modified motif [37]. Here, we found that INTS13-promoted cervical cancer growth is mediated through its effect on hnRNPC expression. We identified hnRNPC as a critical downstream effector, demonstrating that INTS13 regulates its expression in pCCa-1 primary cervical cancer cells. Crucially, the restoration of hnRNPC expression effectively reversed the anti-cancer effects observed upon INTS13 silencing, highlighting hnRNPC as a key mediator in the INTS13-driven oncogenic pathway. This discovery provides a molecular link explaining how INTS13 contributes to the malignant phenotype and offers a potential avenue for therapeutic intervention by targeting this downstream effector.

ZNF384 is a pivotal C2H2-type zinc finger transcription factor instrumental in regulating gene expression through direct DNA binding [38–40]. It plays a significant role in various biological processes, including cancer progression [28, 29, 40]. Critically, recurrent chromosomal translocations involving the ZNF384 gene, which result in fusion proteins with partners such as EWSR1, TAF15, and EP300, are frequently observed in the pathogenesis of acute lymphoblastic leukemia (ALL) [38]. ZNF384 binds to an AT-rich motif in the APOBEC3B (A3B) promoter, and its presence is required for the HPV E6 oncoprotein to activate A3B expression, contributing to HPV-induced carcinogenesis in cervical cancer [41]. Our investigation elucidated an upstream regulatory mechanism for INTS13 overexpression in cervical cancer. Specifically, we identified the transcription factor ZNF384 as a key upstream regulator that directly binds to and positively governs INTS13 expression. This increased binding between ZNF384 and the INTS13 promoter region appears to be a crucial mechanism underlying the observed overexpression of INTS13 in cervical cancer tissues. This finding provides valuable insight into the transcriptional control of INTS13.

In summary, the identification of novel therapeutic targets is of paramount importance for improving the prognosis and treatment outcomes for cervical cancer patients. Our study collectively establishes INTS13 as a crucial precancerous gene in cervical cancer. The consistent overexpression of INTS13 in cervical cancer tissues, its correlation with advanced disease and poorer survival, and its functional validation in vitro and in vivo underscore its potential as a valuable therapeutic target. These findings pave the way for future research into developing targeted therapies against INTS13 or its associated signaling components.

## Supplementary information

Original data (Figure S1)

## Data Availability

All data are available in the Figures and the Supplementary Files.
