# Supplementary material for: Unveiling the ZNF384-INTS13-hnRNPC axis as a therapeutic vulnerability in cervical cancer
Source: Cell Death Dis. 2025 Dec 22;16(1):920. doi: 10.1038/s41419-025-08374-6 (PMC12749084; doi:10.1038/s41419-025-08374-6)

Figure S1. The uncropped blot images associated with the study.

Figure 3.

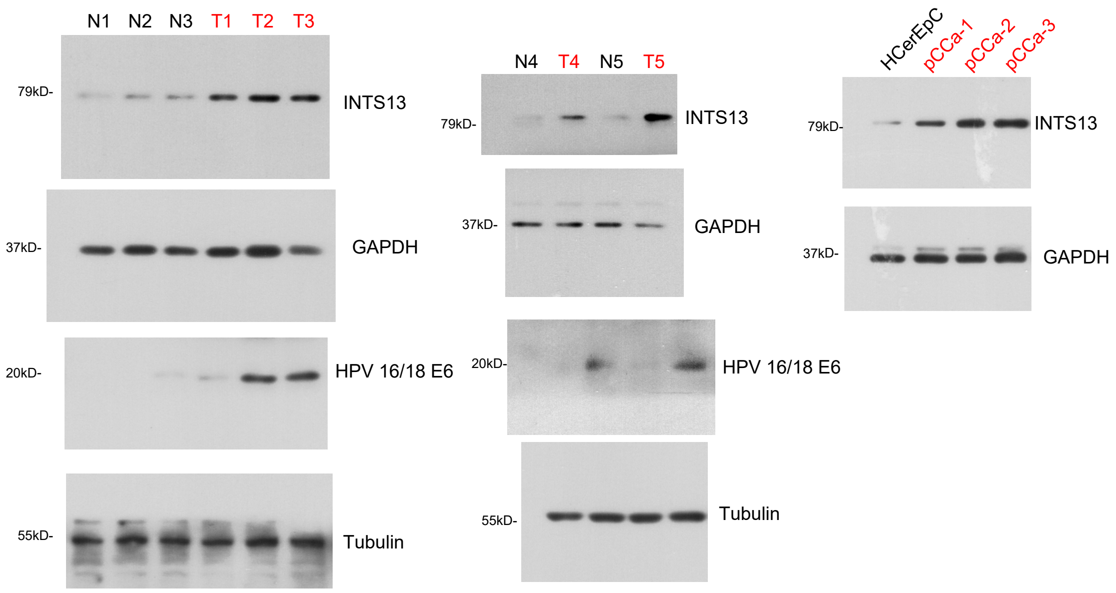

Figure 4.

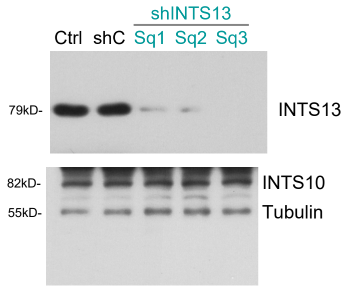

Figure 5.

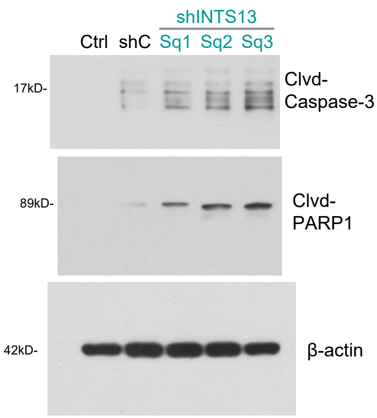

Figure 6.

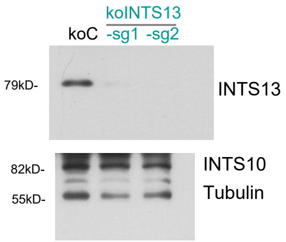

Figure 7.

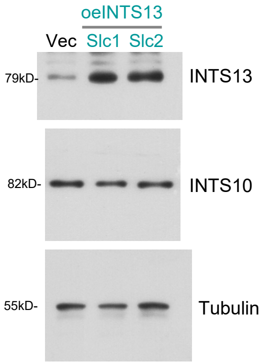

Figure 8.

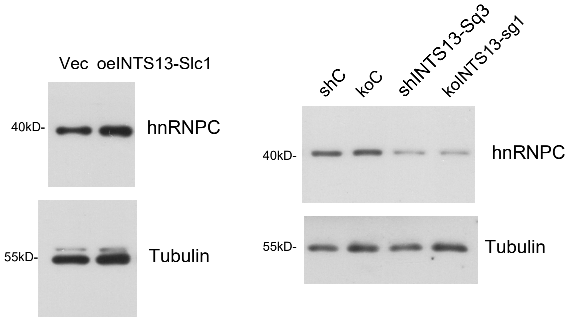

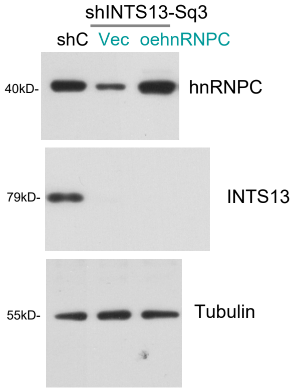

Figure 9.

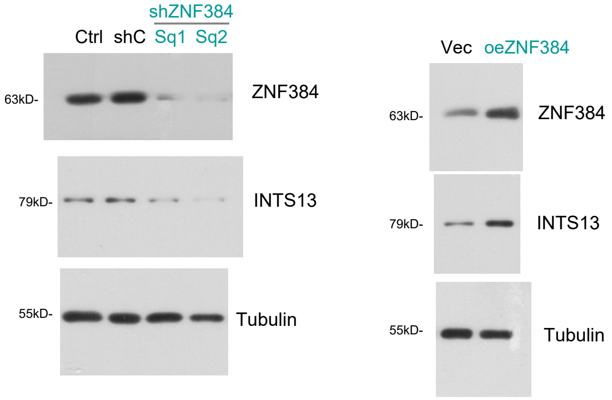

Figure 10.

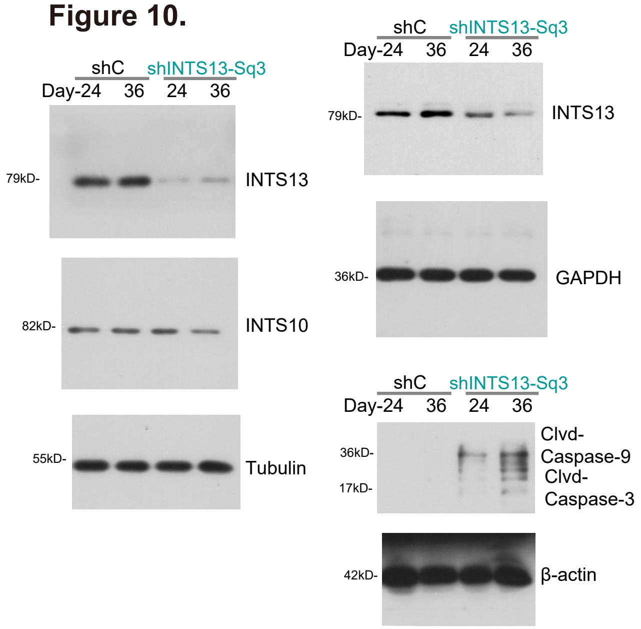

Supplement: Supplementary file 1 — Original data (Figure S1) [file 41419_2025_8374_MOESM1_ESM.pdf]
